# ﻿The genus *Cletocamptus* (Harpacticoida, Canthocamptidae): a reappraisal, with proposal of a new subfamily, a new genus, and a new species

**DOI:** 10.3897/zookeys.1080.71192

**Published:** 2022-01-07

**Authors:** Samuel Gómez, Beatriz Yáñez-Rivera

**Affiliations:** 1 Universidad Nacional Autónoma de México, Instituto de Ciencias del Mar y Limnología, Unidad Académica Mazatlán; Joel Montes Camarena s/n, Mazatlán, 82040, Sinaloa, Mexico Universidad Nacional Autónoma de México Mazatlán Mexico; 2 Consejo Nacional de Ciencia y Tecnología-Centro de Investigación en Alimentación y Desarrollo, Unidad Mazatlán en Acuicultura y Manejo Ambiental; Av. Sábalo Cerritos s/n, Estero del Yugo, Mazatlán, 82112, Sinaloa, Mexico Consejo Nacional de Ciencia y Tecnología-Centro de Investigación en Alimentación y Desarrollo Mazatlán Mexico

**Keywords:** Copepoda, Crustacea, diversity, meiofauna, phylogeny, systematics

## Abstract

A new species of *Cletocamptus* closely related to *C.helobius* was found in sediment samples taken from a polluted estuarine system in north-western Mexico. The genus *Cletocamptus* was relegated to *species incertae sedis* in 1986, and this finding prompted us to evaluate the current taxonomic position of the genus within the Canthocamptidae. The latter has been subdivided in several, seemingly unnatural subfamilies in the past to better understand the relationships between its constituent taxa. In this study we propose a new subfamily, the Cletocamptinae**subfam. nov.** for *Amphibiperita*, *Cletocamptus*, and *Cletocamptoides* gen. nov., defined by the synapomorphic subdistal ventral spinules on the rostrum. The genus *Cletocamptoides***gen. nov.** is proposed for *C.helobius*, *C.merbokensis*, and *C.biushelo***sp. nov.**, and is supported by the ‘cletodid’ shape of the body and the reduced one-segmented endopod of the fourth swimming leg. *Cletocamptus* includes all the other species with long slender spinules on the posterior margin of prosomites and with the sexually modified inner spine on the second endopodal segment of the second swimming leg in the males. *Amphibiperita* retained the primitive female fifth leg with exopod and baseoendopod separated, and the primitive prehensile endopod of the first leg, but is defined by the loss of the antennary exopod. Other (syn)apomorphies are given, and the evolution of the mandibular palp is briefly discussed. Additionally, a diagnosis for the new subfamily, *Cletocamptinae***subfam. nov.**, an amended narrower diagnosis for *Cletocamptus*, the diagnosis for *Cletocamptoides***gen. nov.**, and a phylogenetic analysis supporting the proposal of these new taxa, are given.

## ﻿Introduction

Several canthocamptid species were found in meiofauna samples taken during a short-term study on the effects of organic pollution on the diversity and abundance of harpacticoids. *Cletocamptussinaloensis* Gómez, Fleeger, Rocha-Olivares & Foltz, 2004 was by far the most abundant canthocamptid, followed by Mesochracf.pygmaea (Claus, 1863), but the most intriguing was a form of *Cletocamptus* Schmankevitsch, 1875 closely related to *C.helobius* Fleeger, 1980, the latter known from salt marshes in Louisiana (Fleeger 1980). The genus *Cletocamptus* was erected by Schmankevitsch (1875) for *C.retrogressus* Schmankevitsch, 1875 and the species was allocated in the Cletodidae Scott T., 1904, but Por (1986) removed the genus from the Cletodidae and reallocated it into the Canthocamptidae*incertae sedis* where it stays until today. The find of this material prompted us to analyze and reconsider the taxonomic position of the genus. The taxonomic history of the Canthocamptidae is very complex, and the main approach to understand the relationships amongst its constituent genera has been the proposal of several subfamilies and species groups, e.g., the Rhyncoceratinae Labbé, 1926, the Biarticulata and the Uniarticulata (Chappuis 1929a), the Canthocamptinae Brady, 1880, the Halocanthocamptinae Pesta, 1932, the six species groups of Gurney (1932), the Morariinae Borutzky, 1952, the Epactophaninae Borutzky, 1952, and more recently the *Mesochra* group *sensu*Karaytuğ and Huys (2004), but it seems none of these subfamilies/groups are natural units. In this study, we propose a new subfamily, the Cletocamptinae subfam. nov. for three genera, *Cletocamptus*, *Amphibiperita* Fiers & Rutledge, 1990, and *Cletocamptoides* gen. nov., the latter for *C.helobius*, *C.merbokensis* Gee, 1999, and *Cletocamptoidesbiushelo* sp. nov. The new subfamily is justified by the synapomorphic subdistal ventral spinules of the rostrum. The relationships amongst the new genera are discussed and apomorphies for them and for some species are given.

## ﻿Material and methods

### ﻿Field and laboratory work

Sediment samples were taken at several sampling stations along Urías system (north-western Mexico) (see Gómez 2020: 43, fig. 1) with an Eckman grab (sampling area of 625 cm^2^). Triplicate sediment cores were taken at each sampling site with acrylic corers (sampling area of 24.6 cm^2^) and the upper 3 cm layer of each sample was retrieved and fixed in pure ethanol. Macro- and meiofauna were separated with 500 and 38 µm sieves and meiofauna was extracted through centrifugation with Ludox HS-40 following Burgess (2001) and Rohal et al. (2016) and preserved in pure ethanol. The biological material was sorted at a magnification of 40× using an Olympus SZX12 stereomicroscope equipped with DF PLAPO 1× objective and WHS10× eyepieces, and harpacticoid copepods were stored separately in 1 ml vials with pure ethanol. Illustrations and figures were made from the whole individual and its dissected parts using a Leica DMLB microscope equipped with L PLAN 10× eyepieces, N PLAN 100× oil immersion objective, and drawing tube. The dissected parts were mounted on separate slides using lactophenol as mounting medium. Huys and Boxshall (1991) was followed for general terminology.

**Figure 1.**
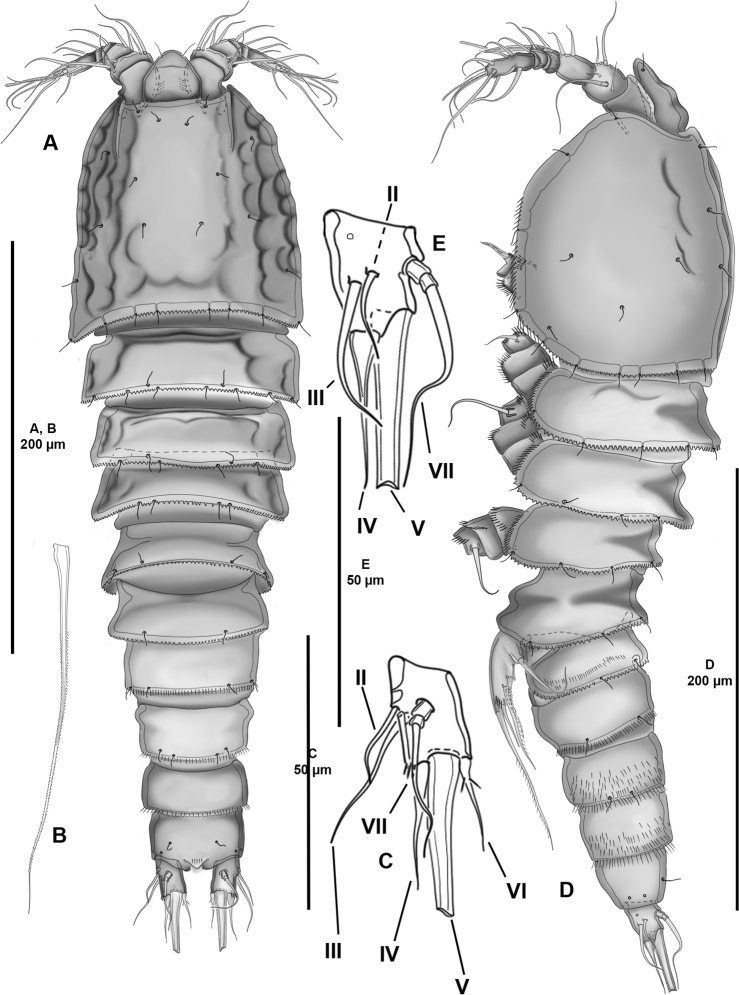
*Cletocamptoidesbiushelo* sp. nov., female holotype **A** habitus, dorsal **B** caudal seta V **C** left caudal ramus, dorsal **D** habitus, lateral **E** left caudal ramus, lateral.

### ﻿Abbreviations used in the text

**BENP** baseoendopod;

**ENP** endopod;

**EXP** exopod;

**EXP (ENP)1 (2, 3)** first (second, third) exopodal (endopodal) segment;

**P1–P6** first to sixth legs.

### ﻿Phylogenetics

A phylogenetic analysis was performed with 39 characters and 36 taxa (Tables 1, 2). The taxa considered in this analysis were: the genus *Cletocamptus* as defined below (species with poor descriptions were excluded), the genus *Cletocamptoides* gen. nov. as defined below, *Amphibiperitaneotropica* Fiers & Rutledge, 1990, and some of the best described species of the subfamilies Canthocamptinae, Hemimesochrinae, and Epactophaninae (see Table 2). We used the Podogennonta as defined by Seifried (2003) as outgroup. Our phylogenetic analysis does not aim at the complete understanding of the relationships amongst all the canthocamptid subfamilies, or amongst the genera and species of Cletocamptinae subfam. nov., and is not to be regarded as definitive, but only as illustrative to support the proposal of Cletocamptinae subfam. nov. and *Cletocamptoides* gen. nov.

**Table 1.** List of characters used for the phylogenetic analysis.

Character	Description
1	Body shape: cletodid, with somitic constrictions between somites (1), non-cletodid, without somitic constrictions between somites (0)
2	Posterior ornament of the cephalothorax: unornamented (0), finely serrated/crenulated (1), coarsely serrated (2), short spinules (3), long spinules (4)
3	Posterior ornament of free prosomites: unornamented (0), finely serrated/crenulated (1), coarsely crenulated (2), coarsely serrated (3), short spinules (4), long spinules (5)
4	Sensilla-bearing socles: absence (0), presence (1)
5	Rostrum: not fused to cephalothorax (0), fused to cephalothorax (1)
6	Rostrum ventral ornament: unornamented (0), spinules or setules (1)
7	Number of segments of the female antennule 1: nine (0), eight (1), seven (2), six (3), five (4), four (5)
8	Number of abexopodal setae on the allobasis of the antenna: two (0), one (1) none (2)
9	Number of segments of the antennary exopod: three (0), two (1), one (2), one- seta (3), absent (4)
10	Number of segments of the mandibular palp: one (0), two or more (1)
11	Ramification of the mandibular palp: biramous (exopod and endopod discernible even if rami fused to basis) (0), monoramous (only endopod discernible even if fused to basis) (1)
12	Number of setae on the mandibular palp: four (0), three (1), two (2), one (3), unarmed (4)
13	Number of setae on the syncoxa of the maxilliped: four (0), one (1), unarmed (2)
14	Shape of P1ENP: prehensile (0), not prehensile (1)
15	Number of segments of P1ENP: three (0), two (1), one (2), represented by spine or seta (3), absent (4)
16	Inner setae on P1 ENP1 when two or three-segmented: present (0), absent (1)
17	Number of segments of P2ENP when present: three (0), two (1), one (2)
18	Inner seta of P2 ENP1 when ENP two- or three-segmented: present (0), absent (1)
19	Inner seta of P2 ENP2 when ENP three-segmented: present (0), absent (1)
20	Number of setae of P2 ENP2 when ENP two-segmented: five (0), four (1), three (2), two (3), one (4), unarmed (5)
21	Number of setae of P2 ENP3 when ENP three-segmented: five (0), four (1), three (2), two (3)
22	Number of setae of P3 EXP3 when EXP three-segmented: eight (0), seven (1), six (2), five (3), four (4)
23	Number of segments of the female P3ENP when present: three (0), two (1), one (2)
24	Inner seta of P3 ENP1 when ENP two- or three-segmented: present (0), absent (1)
25	Number of setae of P3 ENP1 when ENP one-segmented: three (0), two (1)
26	Number of setae of the female P3 ENP2 when ENP two-segmented: six (0), five (1), four (2), three (3), two (4)
27	Number of setae of P4 EXP3 when EXP three-segmented: eight (0), seven (1), six (2), five (3), four (4)
28	Number of segments of P4ENP when present: three (0), two (1), one (2), represented by spine or seta (3)
29	Inner seta of P4 ENP1 when ENP two-segmented: present (0), absent (1)
30	Number of setae of P4 ENP1 when ENP one-segmented: four (0), three (1), two (2), one (3)
31	Number of setae of P4 ENP2 when ENP two-segmented: five (0), four (1), three (2), two (3), one (4)
32	Subdistal spine of P2ENP: present (0), absent (1)
33	Subdistal spine of P3ENP: present (0), absent (1)
34	Female P5BENP/EXP fusion: not fused (0), fused (1)
35	Male rostrum dimorphism: present (1), absent (0)
36	Male P5BENP/EXP fusion: not fused (0), fused (1)
37	Number of setae of male P5ENP lobe: six (0), three (1), two (2), one (3), unarmed (4)
38	Number of segments of male P3: three (0), two (1)
39	Shape of inner apophysis of male P3: arrow-head tip (0), simple (1)

**Table 2.** Character matrix and weights used for the maximum parsimony analysis.

Subfamily	Species	Characters																																						
		1–10										11–20										21–30										31–39								
		1	2	3	4	5	6	7	8	9	0	1	2	3	4	5	6	7	8	9	0	1	2	3	4	5	6	7	8	9	0	1	2	3	4	5	6	7	8	9
Cletocamptinae subfam. nov.	*Amphibiperitaneotropica*	0	3	4	0	0	1	3	1	4	1	1	4	1	0	1	0	1	0	-	2	-	2	1	1	-	2	2	1	1	-	2	1	1	0	1	1	1	1	1
	*Cletocamptoidesbiushelo* sp. nov.	1	1	1	0	0	1	3	0	3	0	1	3	1	1	1	1	1	0	-	2	-	3	1	1	-	3	4	2	-	2	-	0	0	1	?	?	?	?	?
	*Cletocamptoideshelobius* comb. nov.	1	?	?	0	0	1	3	1	3	0	1	3	2	1	1	1	1	0	-	2	-	3	1	1	-	3	4	2	-	3	-	0	0	1	?	1	1	1	1
	*Cletocamptoidesmerbokensis* comb. nov.	1	1	1	1	0	1	3	1	2	0	0	2	2	1	1	1	1	0	-	3	-	3	1	1	-	4	4	2	-	2	-	1	1	1	0	1	1	0	1
	*Cletocamptusalbuquerquensis*	0	4	5	0	0	1	3	0	2	0	1	4	1	1	1	0	1	1	-	2	-	3	1	1	-	1	4	1	1	-	3	0	0	1	1	1	1	1	1
	*Cletocamptusassimilis*	0	4	5	0	0	1	3	0	2	0	1	4	1	1	1	0	1	1	-	2	-	3	1	1	-	3	4	1	1	-	3	0	0	1	?	?	?	?	?
	*Cletocamptusaxi*	0	4	5	0	0	1	3	0	2	0	1	4	1	1	1	0	1	1	-	2	-	3	1	1	-	3	4	1	1	-	3	0	0	1	1	1	1	0	1
	*Cletocamptuscecsurirensis*	0	4	5	0	0	1	3	0	2	0	1	4	1	1	1	0	1	1	-	2	-	3	1	1	-	3	4	1	1	-	3	0	0	1	1	1	1	0	1
	*Cletocamptusconfluens*	0	4	5	0	0	1	3	0	2	0	1	3	1	1	1	0	1	1	-	2	-	3	1	1	-	1	4	1	1	-	3	0	0	1	?	1	1	1	1
	*Cletocamptusdeborahdexterae*	0	4	5	0	0	1	3	0	2	0	1	4	1	1	1	0	1	1	-	2	-	3	1	1	-	3	4	1	1	-	3	0	0	1	1	1	1	0	1
	*Cletocamptusdominicanus*	0	1	1	0	0	1	3	0	2	0	1	4	1	1	1	0	1	1	-	2	-	3	1	1	-	1	4	2	-	2	-	0	0	1	1	1	1	1	1
	*Cletocamptusfourchensis*	0	4	5	0	0	1	3	0	2	0	1	4	1	1	1	1	1	1	-	2	-	3	1	1	-	3	4	1	1	-	3	0	0	1	1	1	1	0	1
	*Cletocamptusgoenchim*	0	4	5	0	0	1	3	0	2	0	1	4	1	1	1	0	1	1	-	2	-	2	1	1	-	1	3	1	1	-	3	0	0	1	1	1	1	0	1
	*Cletocamptusgomezi*	0	4	5	0	1	1	3	1	2	0	1	4	1	1	1	0	1	1	-	1	-	3	1	1	-	1	4	1	1	-	3	0	0	1	?	1	1	0	1
	*Cletocamptuslevis*	0	4	5	0	0	1	3	0	2	0	1	4	1	1	1	0	1	1	-	2	-	3	1	1	-	3	4	1	1	-	3	0	0	1	1	1	1	0	1
	*Cletocamptusnudus*	0	4	5	0	0	1	3	0	2	0	1	4	1	1	1	0	1	1	-	2	-	2	1	1	-	3	3	1	1	-	3	0	0	1	1	1	1	0	1
	*Cletocamptuspilosus*	0	4	5	0	0	1	3	0	2	0	1	4	1	1	1	0	1	1	-	1	-	2	1	1	-	1	3	1	1	-	3	0	0	1	1	1	1	0	1
	*Cletocamptusretrogressus*	0	4	5	0	0	1	3	1	2	1	1	3	1	1	1	1	1	1	-	1	-	2	1	1	-	1	3	1	1	-	3	0	0	1	1	1	1	0	1
	*Cletocamptussamariensis*	0	?	?	0	0	1	3	0	2	0	1	4	1	1	1	0	1	1	-	2	-	2	1	1	-	3	3	1	1	-	3	0	0	1	1	1	1	0	1
	*Cletocamptusschmidti*	0	4	5	0	0	1	3	0	2	0	1	4	1	1	1	0	1	1	-	2	-	2	1	1	-	3	3	1	1	-	3	0	0	1	1	1	1	0	1
	*Cletocamptussinaloensis*	0	4	5	0	0	1	3	0	2	0	1	4	1	1	1	0	1	1	-	2	-	3	1	1	-	3	4	1	1	-	3	0	0	1	1	1	1	0	1
	*Cletocamptusspinulosus*	0	0	5	0	0	1	3	0	2	0	1	4	1	1	1	0	1	1	-	2	-	3	1	1	-	3	4	1	1	-	3	0	0	1	1	1	1	0	1
	*Cletocamptusstimpsoni*	0	4	4	0	0	1	3	0	2	0	1	4	1	1	1	0	1	1	-	2	-	2	1	1	-	1	3	1	1	-	3	0	0	1	1	1	1	0	1
	*Cletocamptustainoi*	0	4	4	0	0	1	3	0	2	0	1	4	1	1	1	0	1	1	-	2	-	3	1	1	-	1	4	1	1	-	3	0	0	1	1	1	1	1	1
	*Cletocamptustertius*	0	4	5	0	0	1	3	0	2	0	1	4	1	1	1	0	1	1	-	2	-	3	1	1	-	3	4	1	1	-	3	0	0	1	1	1	1	0	1
Canthocamptinae	*Canthocamptusincurvisetosus*	0	0	0	0	1	0	1	?	1	?	?	4	1	0	0	0	0	0	0	-	0	1	1	0	-	1	1	1	0	-	0	0	0	0	0	0	2	0	0
	*Canthocamptusodaeensis*	0	0	0	0	1	0	1	?	1	1	0	4	1	0	0	0	0	0	0	-	0	1	1	0	-	1	1	1	0	-	0	0	0	0	0	0	2	0	0
	*Elaphoidellabulgarica*	0	0	0	0	?	?	1	1	2	?	?	?	?	1	0	0	1	0	0	-	-	2	1	0	1	-	2	2	-	1	-	0	0	0	0	0	4	0	1
	*Elaphoidella_ formosanus*	0	0	0	0	1	0	1	?	2	?	?	?	?	1	0	0	1	1	0	-	-	2	1	1	1	-	3	1	0	-	1	0	0	0	0	0	4	0	1
	*Elaphoidellahirsutus*	0	2	3	0	1	0	1	?	2	?	?	?	?	1	0	0	1	0	0	-	-	2	1	1	0	-	2	1	1	-	1	0	0	0	?	?	?	?	?
Hemimesochrinae	*Isthmiocarislaurae*	0	0	0	0	0	0	3	1	2	0	0	3	1	1	1	1	2	-	-	-	-	4	2	-	1	-	4	2	-	3	-	0	0	1	0	0	2	0	0
	*Isthmiocarislongitelson*	0	0	0	0	0	0	3	1	2	1	1	4	1	1	2	-	-	-	-	-	-	5	-	-	-	-	-	-	-	-	-	-	-	1	0	1	3	0	0
	*Itunellaarenaria*	0	0	2	0	0	0	2	1	2	0	0	4	1	1	1	1	2	-	-	-	-	3	2	-	0	-	3	2	-	1	-	0	0	0	0	0	2	1	-
Hepactophaninae	*Stygepactophanes_ occitanus*	0	0	0	0	1	0	2	0	2	0	1	4	2	1	1	1	1	1	-	2	-	4	1	1	-	3	3	2	-	2	-	0	0	0	?	?	?	?	?
	*Epactophanesphilippinus*	0	0	0	0	1	0	2	1	2	0	1	4	2	1	1	1	1	1	-	4	-	3	1	1	-	4	3	2	-	3	-	1	1	0	0	1	4	0	1
Outgroup	Podogennonta	0	0	0	0	?	?	0	?	0	1	0	0	0	0	0	0	0	0	0	-	0	0	0	0	-	-	0	0	0	-	-	?	?	0	?	0	1	0	?
Weights		5	1	3	1	1	A	1	1	1	1	1	1	1	5	1	1	1	5	1	1	1	1	1	1	1	1	1	3	1	1	1	1	1	5	1	1	1	1	1

The maximum parsimony analysis was performed using Phylip ver. 3.697 (Felsenstein 2005) and Bayesian inference with MrBayes ver. 3.2.7a (Huelsenbeck and Ronquist 2001). A Wagner parsimony method with unordered multistates, randomize input order species and weights option was carried out. Missing data were coded as ‘?’, inapplicable states were coded as ‘-’. A consensus tree was obtained from the majority rule and support values were performed from 10,000 bootstrap replicates. For the Bayesian inference, all characters were of equal weights. The default prior distribution of parameters was used for MCMCMC analyses, with one cold chain and three heated chains for 1,000,000 generations and sampled every 100^th^ generation, discarding the first 20% of the burning. Finally, ITOL v5 was used for visualization and editing trees. We used the bioinformatics server Chihuil at Instituto de Ciencias del Mar y Limnología-Universidad Nacional Autómoma de México (**ICML-UNAM**).

In addition to the limited number of canthocamptid taxa used for our phylogenetic analysis, we consulted the available descriptions of 102 species distributed in 45 genera of the different canthocamptid subfamilies for our comparative analysis.

## ﻿Results

### ﻿Taxonomy

**Family Canthocamptidae Brady, 1880**

#### ﻿Subfamily Cletocamptinae subfam. nov.

Taxon classificationAnimaliaHarpacticoidaCanthocamptidae

84E9C87E-B715-522B-B810-5FBD8CB8AB35

http://zoobank.org/E20214E6-38CB-4E00-8082-490FA7FA50A8

##### Type genus.

*Cletocamptus* Schmankevitsch, 1875.

##### Other genera.

*Amphibiperita* Fiers & Rutledge, 1990, *Cletocamptoides* gen. nov.

##### Diagnosis.

Canthocamptidae. Body fusiform, without clear distinction between prosome and urosome. Without nuchal organs on cephalothorax or body somites. Female rostrum distinct, rarely fused to cephalothorax, large and triangular with broad proximal margin; ornamented with ventral (sub)distal spinules or (occasionally) setules and with rounded or (rarely) bilobed tip in both sexes. Cephalothorax and/or prosomites with posterior margins serrated or ornamented with short or long slender spinules; posterior margin of urosomites except for anal somite serrated or with short spinules; posterior margin of cephalothorax and body somites without or (rarely) with cuticular sensillum-bearing socles. Anal operculum without dorsal ornamentation or with transverse row of strong large or short, small spinules; posterior margin unornamented, serrated, or with small or large spinules. Female genital somite and third urosomite separated dorsolaterally, completely fused ventrally forming genital double-somite. Female antennule six-, rarely seven-segmented. Antenna with allobasis, with one or two abexopodal setae (proximal element basal, distal seta endopodal); exopod one-segmented, longer than wide or minute, or absent. Mandibular palp one-segmented, very small and wider than long, or longer than wide; basis and endopod incorporated to basis or (rarely) with basis and endopod distinct (uniramous); exopod absent or (rarely) represented by single seta; when palp uniramous, then basis unarmed or with one seta, endopod with three setae at most; when palp one-segmented and longer than wide, then with two basal and two endopodal setae at most, and exopod (when present) represented by single seta; when palp one-segmented very small and wider than long, then basis and endopod not discernible, with one or two (most probably endopodal) setae, with or without surface (most probably exopodal) seta on coxa. Maxillule with endopod and exopod incorporated to basis; praecoxal arthrite with ventral seta thick and strongly spinulose, or slender and pinnate or smooth. Maxilla with two syncoxal endites; endopod completely incorporated to allobasis. Maxilliped subchelate; syncoxa with one seta; basis unarmed; claw with accessory seta. P1 not prehensile, rarely prehensile; P1–P4EXP three-segmented; female P1–P3ENP two-segmented, P4ENP two- or one-segmented; inner exopodal and endopodal setae with or without comb tip. Female P5EXP and BENP fused, occasionally separated; both baseoendopods of P5 separated. Armature formulae as follows:

**Table:** 

	P1	P2	P3	P4	P5
EXP	0;1;0,1-2,2	0;1;1,2,2	0;1;1-2,2,2	0;0-1;0-2,2,2	♀3-5
					♂3-4
ENP	0-1;1,1-2,1	0-1;0-2,1-2,0-1	♀0;0-2,1-2,0-1	0;0,2,0	♀5-6 (7*)
			♂dimorphic:	or	♂3
			0-1;iap;0,2,0	0,2,0	
			or		
			0-1;iap,2,0		
			or		
			0;iap,1,oap		

iap, inner apophysis; oap, outer apophysis. *The seven-segmented antennule of *C.gravihiatus* (Shen & Sung, 1963) requires confirmation.

Female P6 with one or two setae. Caudal rami with six or seven setae; setae IV and V fused basally or separated.

Sexual dimorphism can be expressed in a) rostrum slenderer in the male, b) the male antennule (chirocer or subchirocer), c) basis of P1 (with or without inner distal process), d) outer spine on P2 ENP2 (thicker and/or shorter than in the female), e) P2EXP and/or P3 and P4EXP (segments longer than in the female, outer spines stronger than in the female, rami curved inwards,), f) shape of P2-P4ENP (segments thicker than in female), g) P3ENP (two- or three-segmented; when three-segmented then inner apophysis on second segment; when two-segmented then inner apophysis medially or subdistally on second segment, occasionally with additional outer apophysis; inner apophysis simple, without arrow-head tip, variable in length; with or without asprothekes on second segment), h) P5 (both legs fused medially or separated; EXP and BENP fused; both legs separated or fused to somite), i) P6 (composed of two lappets articulated to somite or asymmetrical in which case only one leg functional, the other fused to somite; unarmed or (occasionally) with one or two setae), j) caudal rami (longer than in the female).

#### ﻿Genus Cletocamptus

Taxon classificationAnimaliaHarpacticoidaCanthocamptidae

Schmankevitsch, 1875

10CB08EB-30C9-590D-9396-656D50EE9C32

##### Type species.

*Cletocamptusretrogressus* Schmankevitsch, 1875 (type by original designation).

##### Other species.

*Cletocamptusaffinis* Kiefer, 1957, *C.albuquerquensis* (Herrick, 1894), *C.assimilis* Gomez & Gee, 2009, *C.axi* Mielke, 2000, *C.cecsurirensis* Gómez, Scheihing & Labarca, 2007, *C.chappuisi* Gómez, Gerber & Fuentes-Reinés, 2017, *Cletocamptusconfluens* (Schmeil, 1894), *C.deborahdexterae* Gómez, Fleeger, Rocha-Olivares & Foltz, 2004, *C.dominicanus* Kiefer, 1934, *C.feei* (Shen, 1956), *C.fourchensis* Gómez, Fleeger, Rocha-Olivares & Foltz, 2004, *C.goenchim* Gómez, Ingole, Sawant & Singh, 2013, *C.gomezi* Suárez-Morales, Barrera-Moreno & Ciros-Pérez, 2013, *C.gravihiatus* (Shen & Sung, 1963), *C.koreanus* Chang, 2013, *C.levis* Gómez, 2005, *C.mongolicus* Stĕrba, 1968, *C.nudus* Gómez, 2005, *C.pilosus* Gomez & Gee, 2009, *C.samariensis* Fuentes-Reinés, Zoppi de Roa & Torres, 2015, *C.schmidti* Mielke, 2000, *C.sinaloensis* Gómez, Fleeger, Rocha-Olivares & Foltz, 2004, *C.spinulosus* Gomez & Gee, 2009, *C.stimpsoni* Gómez, Fleeger, Rocha-Olivares & Foltz, 2004, *C.tainoi* Gómez, Gerber & Fuentes-Reinés, 2017, *C.tertius* Gomez & Gee, 2009, *C.trichotus* Kiefer, 1929.

**Species *incertae sedis.***

*Marshiabrevicaudata* Herrick, 1894.

##### Species inquirendae.

*Cletocamptusbermudae* Willey, 1930, *C.* cfr. *bicolorsensu*Herbst (1960), *C.brehmi* Kiefer, 1933, *Cletocamptusdeitersi* (Richard, 1897), *C.deitersisensu*Chappuis (1934), *C.deitersisensu*Daday (1902), *C.deitersisensu*Dussart (1974), *C.deitersisensu*Hamond (1973), *C.deitersisensu*Herbst (1960), *C.deitersisensu*Kiefer (1936), *C.deitersisensu*Suárez-Morales et al. (1996), *C.deitersisensu*Tai and Song (1979), *C.ecuadorianus* Löffler, 1963, *C.gabrieli* Löffler, 1961, *C.kummleri* (Delachaux, 1917), *Godotelladadayi* Delachaux, 1917.

##### Doubtful records.

*C.deitersi*: in Apostolov (1984), Brehm (1936, 1965), Chappuis (1936), Dussart and Frutos (1986), Loftus and Reid (2000), Oliveira et al. (1971), Ranga Reddy and Radhakrishna (1979), Ringuelet (1958a, 1958b, 1960, 1962), Ringuelet et al. (1967), Ruber et al. (1994), Sitjar (1988), Zamudio-Valdéz (1991).

##### Diagnosis.

Canthocamptidae: Cletocamptinae. Body fusiform, without clear distinction between prosome and urosome. Without nuchal organs on cephalothorax or body somites. Female rostrum distinct, rarely fused to cephalothorax, large and triangular with broad proximal margin; ornamented with ventral (sub)distal spinules and with rounded tip in both sexes. Posterior margin of cephalothorax and/or prosomites with long and slender, or short spinules, or serrated; posterior margin of urosomites except for anal somite with short spinules or serrated; body somites without cuticular sensillum-bearing socles; anal operculum without dorsal ornamentation or with transverse row of strong, large, or short, small spinules; posterior margin unornamented, or with small or large spinules. Female genital somite and third urosomite separated dorsolaterally, completely fused ventrally forming genital double-somite. Female antennule six-, rarely seven-segmented (the latter reported for *C.gravihiatus* requires confirmation). Antenna with allobasis, with one or two abexopodal setae (proximal element basal, distal seta endopodal); exopod one-segmented and longer than wide, or minute, rarely represented by single seta (the latter reported for *C.chappuisi* requires confirmation). Mandibular palp one-segmented, rarely two-segmented (basis and endopod distinct as in *C.retrogressus*); when one-segmented, then very small and wider than long, or as long as wide (*C.dominicanus*), or longer than wide (*C.confluens*, *C.gomezi*); when palp one-segmented, then basis and endopod not discernible, rarely discernible (represented by single seta as in *C.confluens* and *C.dominicanus*); endopod with two setae, rarely with one single element (*C.pilosus*); exopod absent; with or without surface (most probably exopodal) seta on coxa, the latter present only in some species with a one-segmented palp wider than long, absent in species with palp as long as wide or longer than wide. Maxillule with endopod and exopod incorporated to basis; praecoxal arthrite with ventral seta thick and strongly spinulose, or slender and pinnate or smooth. Maxilla with two syncoxal endites; endopod completely incorporated to allobasis. Maxilliped subchelate; syncoxa with one seta; basis unarmed; claw with accessory seta. P1ENP not prehensile; P1–P4EXP three-segmented; female P1–P3ENP two-segmented, P4ENP two- or one-segmented; inner exopodal and endopodal setae with or without comb tip (see Gómez et al. 2017). Female P5EXP and BENP fused; both baseoendopods of P5 separated. Armature formulae as follows:

**Table:** 

	P1	P2	P3	P4	P5
EXP	0;1;0,1-2,2	0;1;1,2,2	0;1;1-2,2,2	0;0-1;0-1,2,2	♀4-5
					♂3-4
ENP	0-1;1,1,1	0;1-2,1-2,1	♀0;1-2,1-2,1	0;0,2,0	♀5-6 (7^+^)
			♂dimorphic	or	♂3
			0;iap;0,2,0	0,2,0***	
			or		
			0;iap,2,0*		
			or		
			0;iap,1,oap**		

iap, inner apophysis; oap, outer apophysis; * two-segmented with one inner apophysis on second segment in *C.albuquerquensis*, *C.chappuisi*, *C.dominicanus*, *C.tainoi*; ** two segmented, with one inner and one outer apophysis on second segment in *C.confluens*; *** one-segmented in *C.dominicanus*; + the seven setae observed in *C.gravihiatus* requires confirmation

Female P6 with one or two setae. Caudal rami with six or seven setae; setae IV and V fused basally or separated.

Sexual dimorphism expressed in a) rostrum (slenderer than in female), b) the male antennule (subchirocer), c) basis of P1 (with inner distal process), d) outer spine on P2 ENP2 (thicker and/or shorter than in the female), e) P3ENP (three-segmented as in most species, or two-segmented as in *C.albuquerquensis*, *C.chappuisi*, *C.confluens*, *C.dominicanus*, and *C.tainoi*; when three-segmented then inner apophysis on second segment; when two-segmented then inner apophysis medially as in *C.dominicanus*, or subdistally on second segment as in *C.albuquerquensis*, *C.chappuisi*, *C.confluens*, and *C.tainoi*, but occasionally with additional outer apophysis as in *C.confluens*), f) P5 (both legs fused medially as in most species, or separated as in *C.axi*, *C.cecsurirensis*, *C.retrogressus*, and *C.schmidti*; EXP and BENP fused, and both legs articulating with somite, g) P6 (composed of two lappets articulated to somite and unarmed as in most species or, occasionally, with one seta attributable to intraspecific variability as in *C.axi*, *C.schmidti*, *C.sinaloensis*). Additionally, sexual dimorphism can be expressed also (depending on the species) in a) P2EXP and/or P3 and P4EXP (segments longer than in the female, outer spines stronger than in the female, and/or rami curved inwards as in *C.retrogressus*, *C.confluens*, *C.albuquerquensis*, *C.samariensis*, *C.spinulosus*, *C.tainoi*, *C.trichotus*), b) shape of P2–P4ENP (segments thicker than in female as in *C.confluens*), c) caudal rami (longer than in the female).

#### ﻿Genus Amphibiperita

Taxon classificationAnimaliaHarpacticoidaCanthocamptidae

Fiers & Rutledge, 1990

9BF80AB9-5BA0-5103-9F94-8458DA90F020

##### Type species.

*Amphibiperitaneotropica* Fiers & Rutledge, 1990 (type by original designation).

##### Diagnosis.

As in Fiers and Rutledge (1990: 114).

#### ﻿Genus Cletocamptoides gen. nov.

Taxon classificationAnimaliaHarpacticoidaCanthocamptidae

8657F561-3AFC-5EE7-A299-C1FCB378CD4D

http://zoobank.org/21C7CBBC-D8D9-49F5-8A10-DA83FC30E2A1

##### Type species.

*Cletocamptoidesmerbokensis* (Gee, 1999) comb. nov. (= *Cletocamptusmerbokensis* Gee, 1999)

##### Other species.

*Cletocamptoideshelobius* (Fleeger, 1980) comb. nov. (= *Cletocamptushelobius* Fleeger, 1980), *Cletocamptoidesbiushelo* sp. nov.

##### Diagnosis.

Canthocamptidae. Body fusiform, without clear distinction between prosome and urosome; body with somitic constrictions between somites. Without nuchal organs on cephalothorax or body somites. Female rostrum distinct, large, and triangular with broad proximal margin; ornamented with ventral (sub)distal spinules or setules; with rounded or bilobed tip. Cephalothorax and/or prosomites with posterior margins serrated; posterior margin of urosomites except for anal somite serrated; posterior margin of cephalothorax and body somites without or with cuticular sensillum-bearing socles. Anal operculum without dorsal ornamentation or with transverse row of mall spinules; posterior margin unornamented or serrated. Female genital somite and third urosomite separated dorsolaterally, completely fused ventrally forming genital double-somite. Female antennule six-segmented. Antenna with allobasis, with one or two abexopodal setae (proximal element basal, distal seta endopodal); exopod one-segmented and minute, or absent. Mandibular palp one-segmented, longer than wide; endopod incorporated to basis, with two basal and two endopodal setae at most; exopod absent or represented by single seta. Maxillule with endopod and exopod incorporated to basis; praecoxal arthrite with ventral seta thick and strongly spinulose, or slender and pinnate. Maxilla with two syncoxal endites; endopod completely incorporated to allobasis. Maxilliped subchelate; syncoxa unarmed or with one seta; basis unarmed; claw with accessory seta. P1ENP not prehensile; P1–P4EXP three-segmented; female P1–P3ENP two-segmented, P4ENP one-segmented; inner exopodal and endopodal setae with or without comb tip. Female P5EXP and BENP fused, separated by shallow or deep notch; both baseoendopods of P5 separated. Armature formulae as follows:

**Table:** 

	P1	P2	P3	P4	P5
EXP	0;1;0,2,2	0;1;1,2,2	0;1;1,2,2	0;0-1;0,2,2	♀3–4
					♂3–4
ENP	0;1,1,1	0;0-1,1-2,0-1	♀0;0-1,1-2,0-1	0,2,0	♀5–6
			♂dimorphic:		♂3
			0;iap;0,2,0		
			or		
			0;iap,2,0		

iap, inner apophysis; oap, outer apophysis.

Female P6 with two setae. Caudal rami slightly convergent; with six setae; setae IV and V separated; seta IV normal, whip-like, slightly tapering posteriad, or with bulbous base.

Sexual dimorphism can be expressed in a) the male antennule (subchirocer), b) setae on P2 ENP2 (spiniform as in *C.merbokensis* comb. nov.), c) P3ENP (two- or three-segmented; when three-segmented then inner apophysis on second segment (*C.merbokensis* comb. nov.); when two-segmented then inner apophysis subdistally on second segment (*C.helobius* comb. nov.); inner apophysis simple, without arrow-head tip, variable in length), d) P5 (both legs fused medially (*C.merbokensis* comb. nov.) or separated (*C.helobius* comb. nov.); EXP and BENP fused; both legs separated (*C.helobius* comb. nov.) or fused to somite (*C.merbokensis* comb. nov.)), e) P6.

##### Etymology.

The Ancient Greek sufix εἶδος, eîdos, meaning likeness and refers to the resemblance of the new genus with *Cletocamptus*.

#### ﻿ Cletocamptoides biushelo sp. nov.

Taxon classificationAnimaliaHarpacticoidaCanthocamptidae

370F222D-1F58-5CDB-BBCE-948AA5B0CEB8

http://zoobank.org/658B2BB2-1122-454E-B63B-ACC05A76B601

**Figure 2.**
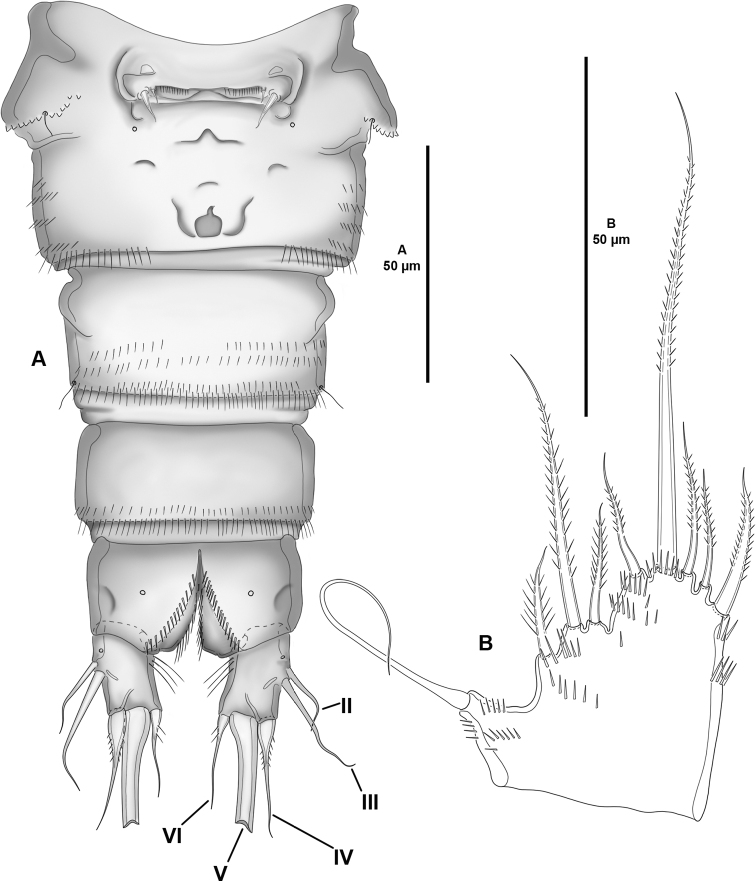
*Cletocamptoidesbiushelo* sp. nov., female holotype **A** urosome, ventral (P5-bearing somite omitted) **B**P5, anterior.

**Figure 3.**
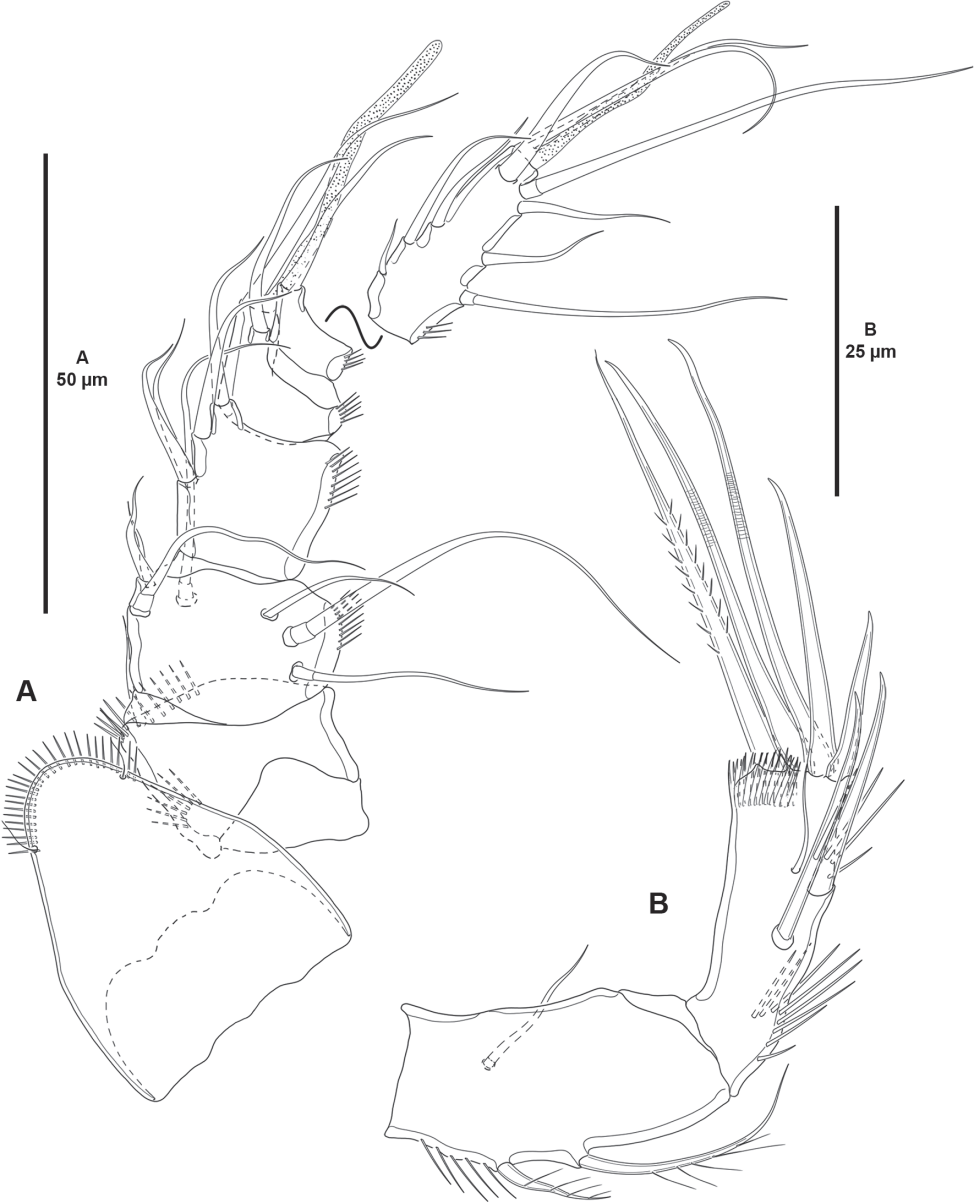
*Cletocamptoidesbiushelo* sp. nov., female holotype **A** rostrum and antennule **B** antenna.

**Figure 4.**
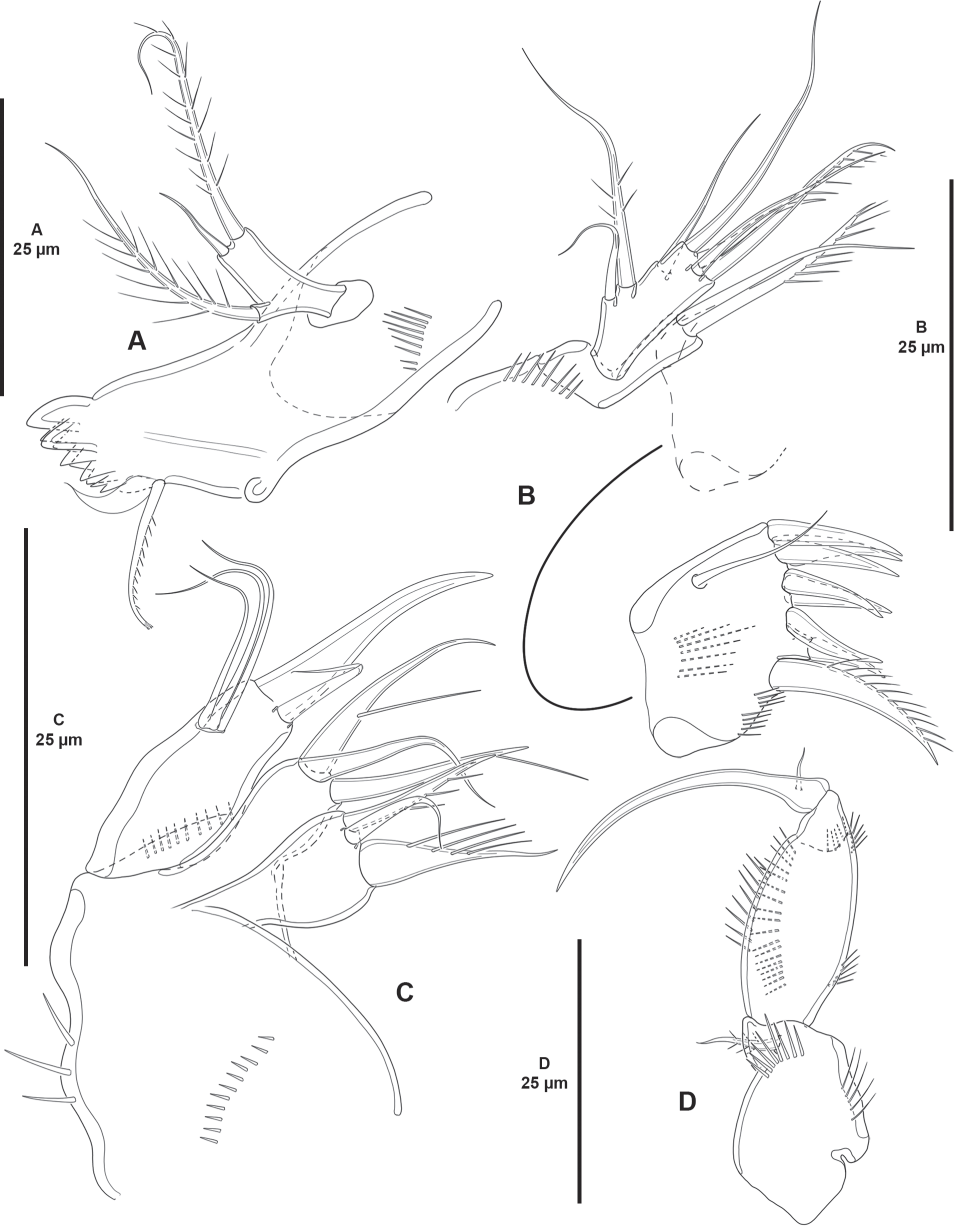
*Cletocamptoidesbiushelo* sp. nov., female holotype **A** mandible **B** maxillule **C** maxilla **D** maxilliped.

**Figure 5.**
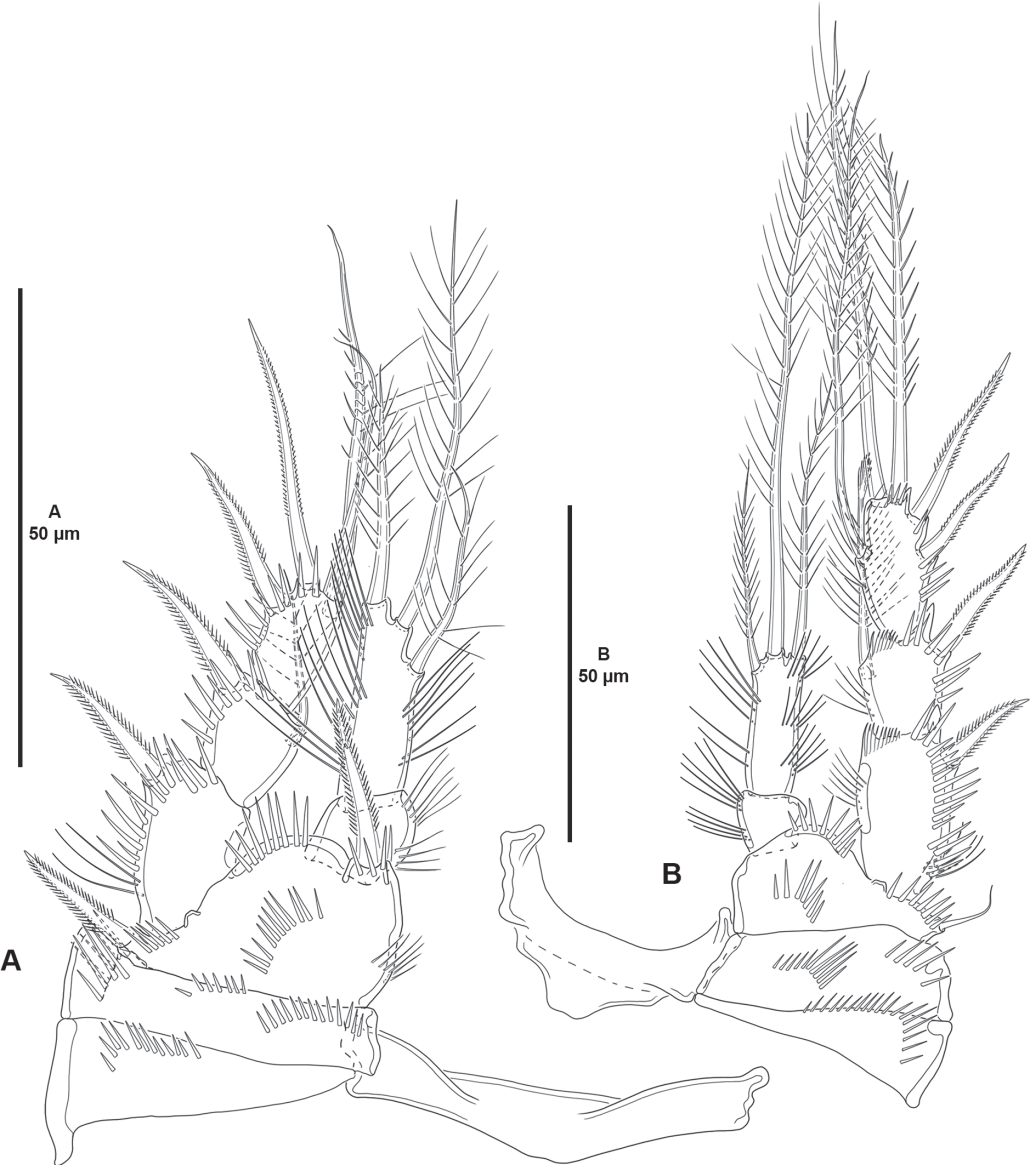
*Cletocamptoidesbiushelo* sp. nov., female holotype **A**P1, anterior **B**P2, anterior.

**Figure 6.**
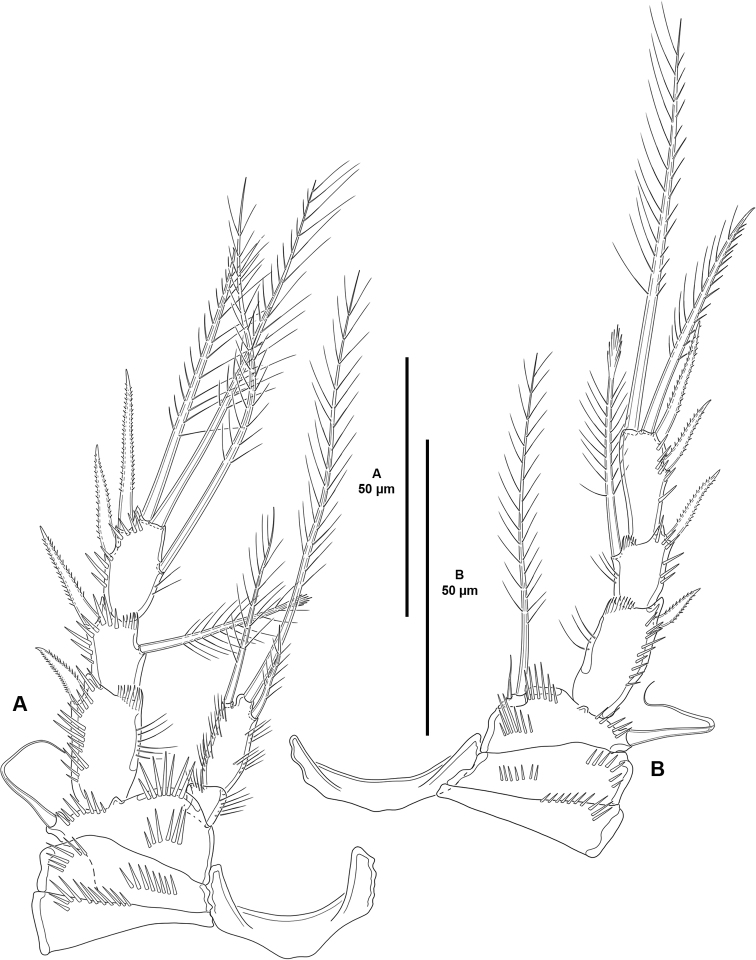
*Cletocamptoidesbiushelo* sp. nov., female holotype **A**P3, anterior **B**P4, anterior.

**Figure 7.**
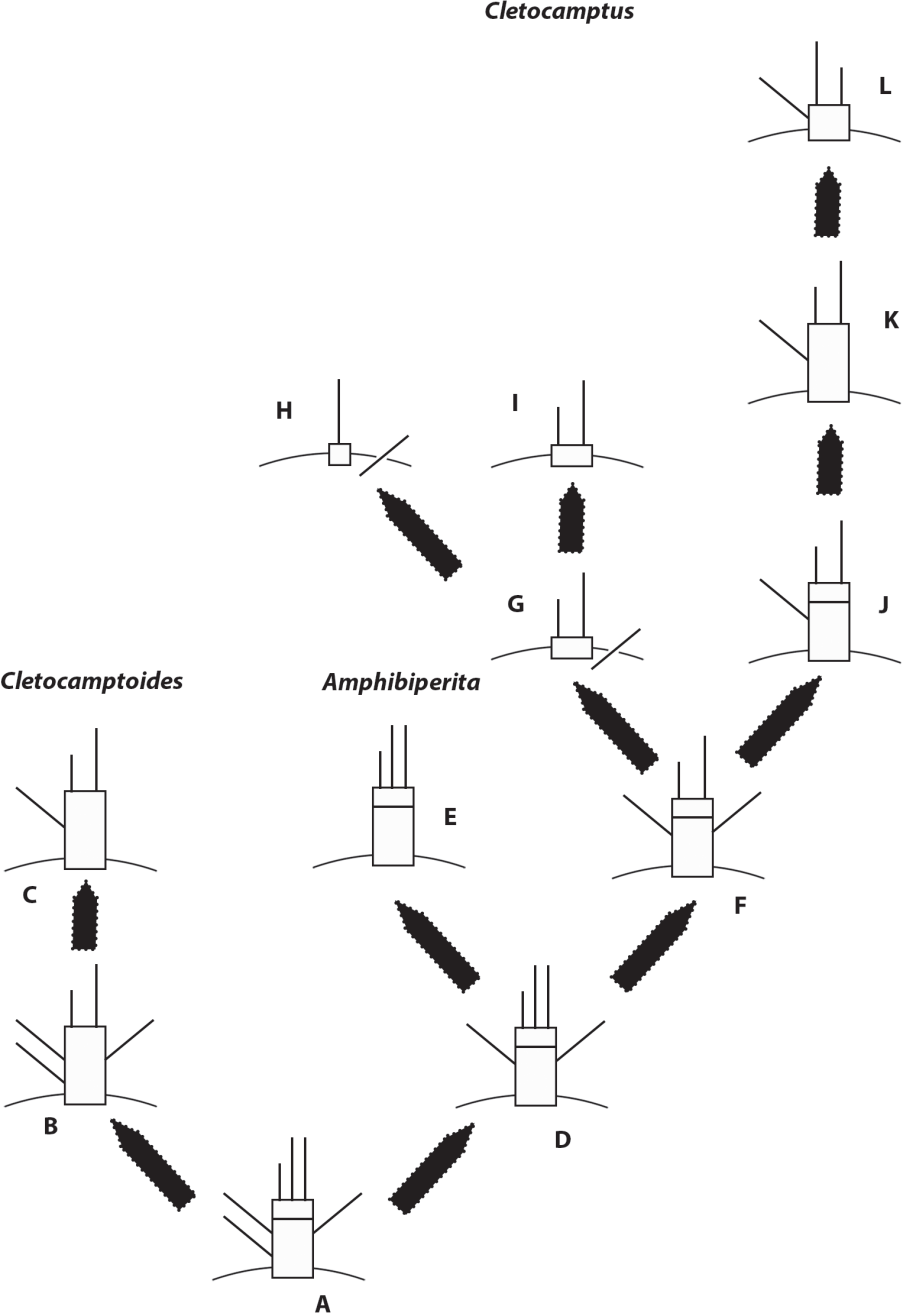
Schematic representation of the evolution of the mandibular palp in Cletocamptinae subfam. nov.

Figs 1 , 2 , 3 , 4 , 5 , 6 , 7

##### Type locality.

Urías estuary, Mazatlán, Sinaloa State, stn. 5 (23.2056°N, 106.3715°W; 0.6 m depth; organic carbon content 0.99%; organic matter content 1.71%; sand 78.61%; clay 6.72%; silt 14.67%) (see also Gómez 2020: 43, fig. 1).

##### Other localities.

Urías estuary, Mazatlán, Sinaloa State, stn. 7 (23.2174°N, 106.3917°W; 3.7 m depth; organic carbon content 5.59%; organic matter content 9.62%; sand 10.78%; clay 37.54%; silt 51.68%) (see also Gómez 2020: 43, fig. 1).

##### Material examined.

Female holotype dissected (ICML-EMUCOP-180119-179); 18 Jan. 2019; S. Gómez leg. One additional female from stn 7 (see above) was used for molecular analyses.

##### Description.

**Female.** Total body length of holotype 411 µm measured from anterior tip of rostrum to posterior margin of caudal rami. Habitus (Fig. 1A, D) semi-cylindrical, progressively tapering posteriad, without clear demarcation between prosome and urosome; body with somitic constrictions between somites; without cuticular sensilla-bearing socles. Prosome (Fig. 1A, D) consisting of cephalothorax, P1-bearing somite fully incorporated to the latter, and three free-pedigerous somites bearing P2–P4. Rostrum (Figs 1A, D, 3A) well-developed, not fused to cephalothorax, triangular, with wide base and rounded tip, with two subdistal sensilla, with row of subdistal spinules ventrally. Cephalothorax with depressions and with sensilla as shown; posterodorsal margin serrated, lateral margin with short slender spinules. P2–P4-bearing somites with posterior sensilla as depicted; posterior margin serrated; lateral margin with short slender spinules. Urosome (Figs 1A, D, 2A) comprising fifth pedigerous somite, genital double-somite, two free abdominal somites, and anal somite. P5-bearing somite largely as in previous somite but with fewer sensilla. Second (genital somite) and third urosomites separated dorsolaterally, fused ventrally forming genital double-somite; first half with posterior margin serrated, posterior half with posterior slender short spinules; both halves with sensilla as shown; anterior half with P6 (see below); posterior half with ventrolateral small slender spinules as depicted. Fourth urosomite largely as preceding somite (second half of genital double-somite), but with more spinules lateroventrally. Fifth somite as previous one dorsolaterally, ventrally with fewer spinular rows. Anal somite slightly wider than long; with small triangular operculum ornamented with small dorsal spinules, flanked by pair of sensilla; ventrally cleft medially, with pair of medial pores, with inner small spinules along inner margin of medial cleft. Caudal rami 1.5 × as long as wide; with inner long slender spinules; with six setae as follows: seta I missing; seta II and III lateral, issuing midway outer margin of ramus, the former proximal to and slightly as long as half the length of the later; seta IV arising at outer distal corner, as long as seta III, with bulbous base; seta V longest, without breaking plane, minutely bipinnate; seta VI issuing at inner distal corner, slightly shorter than seta IV, with two proximal spinules as shown, with bulbous base; dorsal seta VII located at the center of ramus, as long as seta III, tri-articulated at its base.

Antennule (Fig. 3A) six-segmented; first segment with two inner spinular rows, remaining segments with outer spinular row as depicted; all setae smooth. Armature formula as follows: 1[1], 2[7], 3[4], 4[1+(1+ae)], 5[1], 6[8+(2+ae)].

Antenna (Fig. 3B) with allobasis ornamented with short longitudinal row of spinules proximally, armed with two abexopodal setae (one basal, one endopodal). Exopod one-segmented, minute, with one seta. Free endopodal segment as long as allobasis, with inner spinules proximally and subdistally, and with outer subdistal frill; with two inner spines and one slender seta medially, distally with two inner spines, two medial geniculate setae, and one outer spinulose element.

Mandible (Fig. 4A) with well-developed coxa ornamented with short spinular row as shown; gnathobase well-developed, with bi- and unicuspid teeth distally, and one thick bulbous element, and one ventral pinnate seta. Palp one-segmented, with three setae (one basal, two endopodal).

Maxillule (Fig. 4B) with robust praecoxa ornamented with spinules at base of coxal endite, and medially and ventrally on arthrite, the latter with one surface seta, seven distal spines and one ventral strong unipinnate seta. Coxal endite with two setae. Endopod and exopod incorporated to basis, the latter with four, endopod with one, exopod with two setae.

Maxilla (Fig. 4C) with syncoxa ornamented with medial and distal rows of small spinules, and with few larger outer spinules; with two endites bearing three setae each. Allobasis drawn out into claw accompanied by one seta. Endopod completely incorporated to basis, represented by three setae.

Maxilliped (Fig. 4D) subchelate. Syncoxa with spinules as shown, with one subdistal seta. Basis with one anterior and one posterior spinular row, with outer spinules proximally and subdistally. Endopod drawn out into curved claw with minute accessory seta.

P1 (Fig. 5A) with elongate bare intercoxal sclerite. Praecoxa triangular, with medial subdistal row of spinules. Coxa rectangular, wider than long, with two medial transverse rows of spinules as shown, and one posterior and one anterior short row of larger spinules close to outer margin. Basis with anterior spinules medially, on outer margin, at base of inner and outer spines, and between rami. Exopod three-segmented, situated at a lower level than the endopod, reaching tip of ENP2; segments with outer and subdistal spinules as shown; EXP1 without, EXP2 with inner seta; EXP3 with two outer spines and two distal setae of which innermost geniculate. Endopod two segmented, not prehensile; segments with inner and outer long slender spinules as depicted; ENP1 unarmed; ENP2 with three setae.

P2–P3 (Figs 5B, 6A) with elongate bare intercoxal sclerite. Praecoxa triangular, with transverse row of spinules as shown. Coxa rectangular, wider than long; with one medial and one outer spinular row. Basis with spinules medially, at base of endopod, and between rami; with outer seta (of P2 visibly shorter than in P3). Exopod three-segmented, situated at a lower level than endopod; spinular ornamentation of segments as shown; EXP1 and EXP2 with, EXP3 without inner distal frill; EXP1 without, EXP2 with inner seta with comb tip; EXP3 with one inner element without comb tip, two distal setae, and two outer spines. Endopod two-segmented; segments with spinular ornamentation as shown; ENP1 small, as long as wide, unarmed; ENP2 elongated, with three setae.

P4 (Fig. 6B) with intercoxal sclerite, praecoxa, coxa and basis as in P3. Exopod largely as in P3 except for two apical setae, and two outer spines on EXP3. Endopod one-segmented, minute, wider than long; with two setae of which innermost reduced.

**Table:** 

	P1	P2	P3	P4	P5
Exopod	0,1,022	0,1,122	0,1,122	0,1,022	3
Endopod	0,210	0,111	0,111	020	5

P1–P5 armature formulae as follows:

P5 (Fig. 2B) with baseoendopod and exopod fused, rami separated by shallow notch; spinular ornamentation as shown. Baseoendopod with outer seta arising from short setophore; endopodal lobe with five setae. Exopod with three setae.

Genital field (Fig. 2A) with median copulatory pore on second half of genital double-somite; each P6 represented by two setae.

**Male.** Unknown.

##### Etymology.

The specific epithet is an anagram for “helobius” and refers to the close relationship between the Mexican new species and *C.helobius* comb. nov.

### ﻿Phylogenetics

The selected characters (Table 1) in the matrix (Table 2) integrate the main attributes of canthocamptids regarding the body shape (character 1), posterior ornament of the cephalothorax and prosomites (characters 2 and 3), presence of cuticular sensillum-bearing socles (character 4), shape and ornamentations of the rostrum (characters 5 and 6), segmentation of the female antennule (character 7), armature complement of the antennary allobasis and segmentation of the antennary exopod (characters 8 and 9), segmentation, shape, and armature complement of the mandibular palp (characters 10–12), armature complement of the syncoxa of the maxilliped (character 13), several features of P1–P5 (characters 14–34), and male dimorphism (characters 35–39).

**Figure 8.**
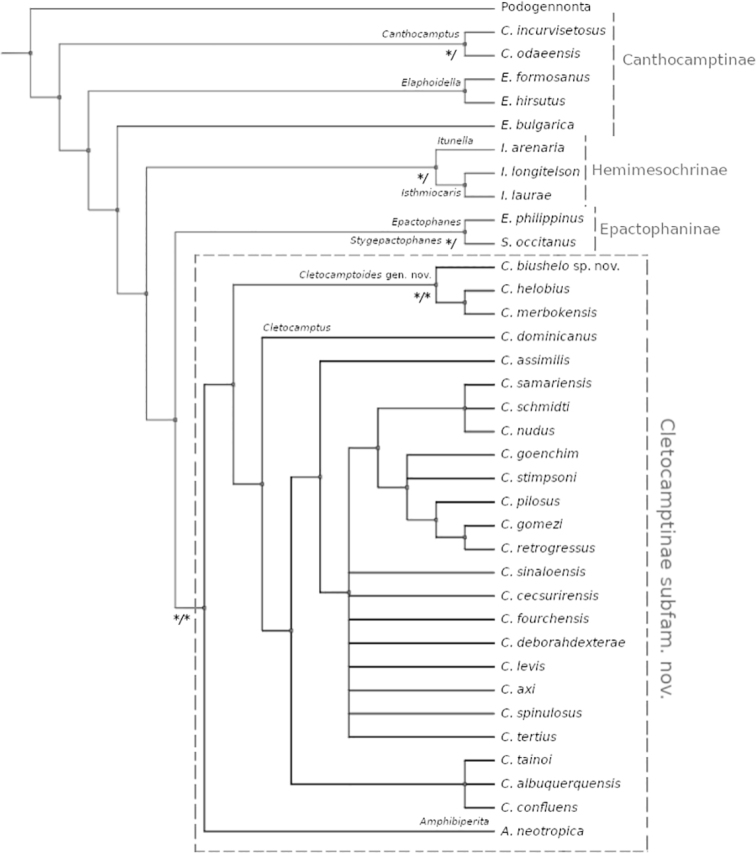
Phylogenetic hypothesis showing the relative position of Cletocamptinae subfam. nov. and *Cletocamptoides* gen. nov. Bootstrap (boot) and BI posterior probabilities (pp) represented as boot/pp. * Nodes of high support, boot > 90%; pp > 0.95.

The heuristic search yielded nine equally most parsimonious trees of 220 steps, and consistency index of 0.39. The extended majority rule consensus tree is shown in Figure 8. The results show the monophyly of Cletocamptinae subfam. nov. and *Cletocamptoides* gen. nov. which are well supported clades with high bootstrap values. The Bayesian inference analysis recognized and supported the monophyly of Cletocamptinae subfam. nov. and *Cletocamptoides* gen. nov. Our Bayesian results indicate that *Amphibiperita* is nested within *Cletocamptus*, rendering the relationships between the genus *Cletocamptus* and *Amphibiperita* unresolved. However, we maintained the genus *Amphibiperita* separated from *Cletocamptus* due the maximum parsimony support as well as their unique characteristics (see below).

## ﻿Discussion

### ﻿Historical background and identification of the problem

The family Canthocamptidae was created and diagnosed by Brady (1880: 47) for *Canthocamptus* Westwood, 1836, *Mesochra* Boeck, 1865, *Attheyella* Brady, 1880, *Tetragoniceps* Brady & Robertson, 1876, *Diosaccus* Boeck, 1872, *Laophonte* Philippi, 1840, *Normanella* Brady, 1880, *Cletodes* Brady, 1872, and *Enhydrosoma* Boeck, 1872. The subfamily was subsequently raised to family level by Sars (1906: 193). Sars (1911: 418) foresaw the separation of *Pteropsyllus* Scott T., 1906a, *Evansula* Scott T., 1906b, *Tetragoniceps*, and *Leptastacus* Scott T., 1906a from the Canthocamptidae to a different family. Several groups/subfamilies have been proposed in the past as an effort to disentangle the complicated phylogenetic relationships amongst the canthocamptid genera.

Labbé (1926) proposed a new subfamily of Canthocamptidae, the Rhyncoceratinae, and one year later, Labbé (1927) gave a more detailed description of the species within that subfamily with additional figures. Labbé’s (1926; 1927) works were plagued with taxonomical errors, wrong identifications, and descriptions, and were harshly criticized by Gurney (1927) and Lang (1948) who finally disposed of the Rhyncoceratinae. As far as we are aware, the subfamily Rhyncoceratinae was never mentioned afterwards.

In a preliminary note on the revision of the freshwater genus *Canthocamptus*, Chappuis (1929a) subdivided the genus into several new genera distributed in two groups based on the one- or two-segmented condition of the antennary exopod, the Biarticulata for *Canthocamptus*, *Bryocamptus* Chappuis, 1929a and *Echinocamptus* Chappuis, 1929a (= Bryocamptus (Echinocamptus) Chappuis, 1929a), and the Uniarticulata for *Attheyella* and *Elaphoidella* Chappuis, 1929a. That same year, Chappuis (1929b) abandoned that system. Sixteen marine, one brackish, and nine freshwater genera were known at the time of Chappuis’ (1929b) paper, but he could not inspect but freshwater material (Chappuis 1929b). However, based on the available literature, he noted that the marine and brackish genera were far different (for a list of marine and freshwater genera allocated into the Canthocamptidae by that time, see Monard 1927) and proposed and diagnosed the subfamily Canthocamptinae for freshwater canthocamptids: *Canthocamptus*, *Paracamptus* Chappuis, 1929b (= *Pesceus* Özdikmen, 2008), *Bryocamptus*, *Maraenobiotus* Mrázek, 1893, *Hypocamptus* Chappuis, 1929b, *Echinocamptus* (= Bryocamptus (Echinocamptus)), *Ceuthonectes* Chappuis, 1924, *Moraria* Scott T. & Scott A., 1893, *Attheyella*, *Elaphoidella*, *Epactophanes* Mrázek, 1893, and several species incertae sedis (Chappuis 1929b: 495–496). Pesta (1932) believed that the morphological variability of the Canthocamptidae could be better understood by grouping its genera into two subfamilies. For this, he (Pesta 1932) proposed and diagnosed the subfamily Halocanthocamptinae for those genera inhabiting coastal marine and brackish waters, as well as continental saline habitats: *Mesochra* Boeck, 1865, *Paramesochra* Scott T., 1892, *Remanea* Klie, 1929, *Leptomesochra* Sars, 1911, *Evansula*, and *Leptastacus* (Pesta 1932: 79), and the subfamily Canthocamptinae Brady, 1880 for freshwater forms: *Nitocrella* Chappuis, 1924, *Canthocamptus*, *Hypocamptus*, *Maraenobiotus*, *Moraria*, *Epactophanes*, *Elaphoidella*, *Attheyella*, *Paracamptus*, *Echinocamptus*, and *Bryocamptus* (Pesta 1932: 91–92). Monard (1935) expressed some doubts about the naturalness of Pesta’s (1932) scheme but believed that it was, at least, practical. Gurney (1932) did not accept the concept of the Ameiridae and subdivided the Canthocamptidae into six groups, two of which (the first and the third) were included by Lang (1936) in his list of ameirid genera. The other groups of Gurney (1932) are a) *Tetragoniceps* for *Tetragoniceps*, *Phyllopodopsyllus* Scott T., 1906a, *Pteropsyllus*, *Paramesochra*, *Leptopsyllus* Scott T., 1894, and *Diagoniceps* Willey, 1930, b) *Evansula* for *Evansula*, *Leptastacus*, and *Leptopontia* Scott T., 1902, c) *Cletomesochra* for *Cletomesochra* Sars, 1920 (= *Heteropsyllus* Scott T., 1894), *Hemimesochra* Sars, 1920, *Nannomesochra* Gurney, 1932, *Leptomesochra*, *Pseudomesochra* Scott T., 1902, and *Paramesochra*, and d) *Canthocamptus* for the freshwater canthocamptids and *Mesochra*.

In a preliminary note, Lang (1944) removed *Paramesochra* from the Canthocamptidae to his newly created family Paramesochridae Lang, 1944, and proposed a new family, Tetragonicepsidae Lang, 1944 (whose spelling was subsequently corrected to Tetragonicipitidae) for *Tetragoniceps*, *Pteropsyllus*, *Diagoniceps*, *Phyllopodopsyllus* and *Paraphyllopodopsyllus* Lang, 1944 (= *Phyllopodopsyllus*). Lang (1948) believed that the Canthocamptidae and the Cletodidae bear a sister group relationship and that the difficulties for the separation of both families are the result of poor descriptions of freshwater canthocamptids. He (Lang 1948) rejected all the subdivisions of the Canthocamptidae pending a thorough review of the family, and included into this family the following genera: *Hypocamptus*, *Maraenobiotus*, *Morariopsis* Borutzky, 1931, *Itunella* Brady, 1896, *Orthopsyllus* Brady & Robertson, 1873, *Ceuthonectes*, *Spelaeocamptus* Chappuis, 1933, *Attheyella*, *Canthocamptus*, *Bryocamptus*, *Nannomesochra*, *Mesochra*, *Echinocamptus*, *Moraria*, *Paracamptus*, *Antarctobiotus* Chappuis, 1930, *Afrocamptus* Chappuis, 1932, *Epactophanes*, and *Elaphoidella*.

By the early 1950’s, Borutzky (1952) re-diagnosed the Canthocamptidae and the subfamilies Halocanthocamptinae, with *Mesochra* and “the genera most closely related to it “ (Borutzky 1952: 124), and Canthocamptinae with *Canthocamptus*, *Paracamptus*, *Bryocamptus*, *Arcticocamptus* Chappuis, 1929b (= Bryocamptus (Arcticocamptus) Chappuis, 1929b), *Echinocamptus* (= Bryocamptus (Echinocamptus)), *Maraenobiotus*, *Hypocamptus*, *Attheyella*, *Elaphoidella*, and *Spelaeocamptus*, and added the Morariinae for *Ceuthonectes*, *Moraria*, *Morariopsis* and some species incertae sedis, and the Epactophaninae for *Epactophanes*. He (Borutzky 1952) diagnosed these four subfamilies based on the structure of the female genital field and on the structure of the male P3ENP. Later on, Por (1986: 423) proposed and (re)diagnosed the subfamily Hemimesochrinae Por, 1986 for those genera which, despite having a non-prehensile P1ENP, were left into the Canthocamptidae in Lang (1948); these genera are: *Nannomesochra*, *Hemimesochra*, *Heteropsyllus*, *Mesopsyllus* Por, 1960, *Poria* Lang, 1965 (= *Hanikraia* Huys, 2009), and *Dahlakia* Por, 1986 (= *Dahlakocamptus* Huys, 2009). Additionally, Por (1986) relegated to incertae sedis the genera *Cletocamptus* and *Parepactophanes* Kunz, 1935, as well as *Hemimesochrarapiens* Becker, 1979 (= *Perucamptusrapiens* (Becker, 1979)), and *Heteropsyllusserratus* Schriever, 1983. One year later, Hamond (1987) suggested to consider no less than 18 taxa as subgenera of *Canthocamptus* s. lat. “until all their respective type species, and as many others as possible from all over the world, can be studied again by modern standards “… “as part of a world revision“ (Hamond 1987: 1027; but see also Wells 2007).

With 864 species distributed in 58 valid genera (WoRMS 2021), the Canthocamptidae is currently one of the most species-rich families within Harpacticoida. The Canthocamptidae is not a natural assemblage and could be divided into several families (Huys et al. 1996; Gómez Noguera and Fiers 1997; Boxshall and Halsey 2004; Huys and Conroy-Dalton 2006). The taxonomy and the phylogenetic relationships are immersed in chaos, and the different canthocamptid subfamilies are seldom mentioned in the recent literature probably because the genera described more recently are difficult to attribute to any of the existing subfamilies, and if forced, the subfamilial diagnoses have often to be widened more to include, probably unrelated taxa, exacerbating the already complicated systematics of the group. This problem is also common at the genus level, and is evident in *Hemimesochra*, which is a heterogeneous grouping of distantly related taxa (see Huys and Thistle 1989). The complexity of the Canthocamptidae is also fuelled by the weak boundaries between the Canthocamptidae and the Cletodidae and their respective genera. Huys and Thistle (1989) agreed with Por (1986) in that the dismantling of the Cletodidae*sensu*Lang (1944, 1948) was a necessary step, but did not accept Por’s (1968) view of taking the “variability within the Cletodidae, and the notion of genus itself “… “in a much wider sense than in any other harpacticoid family“ (Por 1968: 46). Huys and Thistle’s (1989) reasoning is adopted here. Huys and Thistle (1989) succeeded in narrowing the generic diagnosis of *Hemimesochra* by distributing its species in *Boreolimella* Huys & Thistle, 1989, *Perucamptus* Huys & Thistle, 1989, *Carolinicola* Huys & Thistle, 1989, *Mesopsyllus*, and *Pusillargillus* Huys & Thistle, 1989, leaving *H.clavularis* Sars, 1920 as the type and only species of *Hemimesochra*. They (Huys and Thistle 1989) concluded that *Bathycamptus* Huys & Thistle, 1989, *Psammocamptus* Mielke, 1975 and *Boreolimella* are phylogenetically related, being *Bathycamptus* the sister taxon of *Psammocamptus*, that *Boreolimella* is probably the sister taxon of the *Bathycamptus*-*Psammocamptus* lineage, and that *Mesopsyllus* could be a potential relative of this genus group.

George and Schminke (2003) noted that *Isthmiocaris* George & Schminke, 2003 could well be related to *Bathycamptus* and *Itunella*. Later on, Huys and Kihara (2010) removed *Metahuntemannia* Smirnov, 1946 and *Pottekia* Huys, 2009 from the Nannopodidae Brady, 1880 and found that these two genera are probably related to *Psammocamptus*, *Bathycamptus*, *Perucamptus*, and *Isthmiocaris*. However, as far as we are aware, no apomorphies have been found so far for the Hemimesochrinae. The same applies for the other canthocamptid subfamilies.

Karaytuğ and Huys (2004) proposed a practical, not necessarily phylogenetic, group of marine and brackish canthocamptids, the *Mesochra* group, in which the male P6 is represented by unarmed membranous flaps, and the female P6 is represented by one to three setae. Following Karaytuğ and Huys (2004), this species group includes species currently allocated into the Canthocamptinae and Hemimesochrinae, and two species incertae sedis; these are: *Mesochra* and *Amphibiperita* (Canthocamptinae), and *Psammocamptus*, *Bathycamptus*, *Mesopsyllus*, *Isthmiocaris*, *Hemimesochra*, *Poria*, *Perucamptus*, *Pusillargillus* (Hemimesochrinae), and *Taurocletodes* Kunz, 1975, and *Parepactophanes* (Canthocamptidae incertae sedis). The genus *Mesochra* is not a natural unit and is most probably polyphyletic (Gómez Noguera and Fiers 1997; Karaytuğ and Huys 2004), but if Karaytuğ and Huys’ (2004) assumption is correct, this might reflect the artificial nature of the canthocamptid subfamilies.

More recently, George (2020) contributed with important changes to the family Cletodidae*sensu*Por (1986), which has been regarded in the past as the sister taxon of the Canthocamptidae. He (George 2020) proposed the CletodoideaBowman and Abele (1982) for some Ancorabolidae Sars, 1909 *sensu*George (2020) and Cletodidae for which he detected 19 autapomorphies (George 2020). George (2020) also gave an amended diagnosis for the Cletodidae, which he extended to include several genera that belonged to the Ancorabolidae, and proposed a new subfamily, the Cletodinae Scott T., 1904 for the ancorabolid *Ceratonotus* group *sensu*Conroy-Dalton (2001) and *Cletodes*, defined by seven autapomorphies (George 2020). For the Cletodidae, he (George 2020) detected a set of five autapomorphies: a) P1ENP not prehensile, P1 ENP1 dwarfed; b) P1 ENP2 elongate, at least as long as ENP1; c) the medial apical geniculate seta of the Cletodoidea (seta 5-en in George 2020: 470, fig. 3C) reverted into a bipinnate or plumose seta (5-en in George 2020: 470, fig. 3D) in Cletodidae; d) the outer distal claw-like element on the P1 ENP2 in the Cletodoidea (1-en in George 2020: 470, fig. 3C) reverted into a spine (1-en in George 2020: 470, fig. 3D) in Cletodidae; and e) loss of the accompanying seta on the maxillipedal claw.

The complexity of the Canthocamptidae is evident and it is due to bad and incomplete descriptions (Hamond 1987). Hamond (1987) proposed the revision of 18 taxa as part of a world revision using modern standards. Wells (2007) commented positively on Hamond’s (1987) proposal but at the same time he foresaw that this would not find ready support due to the massive effort that this would imply. Some authors have contributed to our understanding of the intergeneric relationships within isolated subfamilies. Huys and Thistle (1989), George and Schminke (2003), and Huys and Kihara (2010) have proved, at least partially, that the subfamily Hemimesochrinae is a compact unit, not so the other canthocamptid subfamilies. In our opinion, the establishment of well-defined taxonomic taxa (subfamilies, genera, species groups, etc.) is an obligate step towards a world revision of the family. The relationships between the Canthocamptinae and other genera outside that subfamily are still uncertain, and the proposal of a new subfamily, Cletocamptinae subfam. nov., for a well-defined, seemingly monophyletic group of genera (*Cletocamptus*, *Amphibiperita*, and *Cletocamptoides* gen. nov.) as well as the narrowing of the diagnosis for *Cletocamptus*, are justified and follow the same reasoning as explained below.

### ﻿Reconstruction of the common ancestor of the Cletocamptinae subfam. nov.

The high morphological diversity of the Canthocamptidae makes difficult the detection of (aut)apomorphies to substantiate the establishment and proposal of monophyletic taxa. In this sense, the role of weighted characters for the maximum parsimony approach was essential and allowed high support. The genus *Cletocamptus* as known before the present study, seems to be composed of two different lineages, the *merbokensis*-*helobius*-*biushelo*-lineage (mhb-lineage; for *C.merbokensis*, *C.helobius*, and *C.biushelo* sp. nov.) and the *retrogressus*-lineage (for the rest of the species of *Cletocamptus*). The split of *Cletocamptus* into two genera is proposed in this study: *Cletocamptoides* gen. nov. for the mhb-lineage, and *Cletocamptus* for the *retrogressus*-lineage.

The reconstruction of the hypothetical ancestor of the Cletocamptinae subfam. nov. is necessary to justify the (aut)(syn)apomorphies for the three genera within the new subfamily. The hypothetical ancestor was reconstructed based on plesiomorphic and (aut)apomorphic states of a selected set of characters, as essential to explain our hypotheses and phylogenetic reasoning.

Most canthocamptids possess a body without somitic constrictions between somites, and few species, e.g., *C.merbokensis* comb. nov., *C.helobius* comb. nov., *C.biushelo* sp. nov., possess a body with somitic constrictions between somites conferring the body a cletodid appearance. The latter is regarded here as the derived (synapomorphic) condition for the mhb-lineage. The ancestral Cletocamptinae subfam. nov. is hypothesized to lack somitic constrictions between somites, tapering gradually posteriad. The latter is considered as the primitive condition and is present in the *retrogressus*-lineage.

The posterior margin of body somites of most genera of the Canthocamptidae is plain, without any ornamentation, including one species of Cletocamptinae subfam. nov., *C.chappuisi*, but its condition requires confirmation; it is finely or deeply serrated in some genera, e.g., *Antrocamptus* Chappuis, 1957, *Attheyella*, *Canthocamptus*, *Elaphoidella*, *Moraria*, *Paramoriaropsis* Brancelj, 1991, *Spelaeocamptus*, and *C.dominicanus*; it is ornamented with short spinules, e.g., *Attheyella*, and *C.albuquerquensis* and *C.tainoi*, or with long slender spinules, e.g., *Cletocamptus* as defined here. It is assumed that the plain condition of the posterior margin of body somites is the most primitive within the Canthocamptidae. Species of some genera developed serrate posterior margins that eventually developed into short spinules. The ancestral Cletocamptinae subfam. nov. is hypothesized to have retained the finely serrate posterior margins present in *Cletocamptoides* gen. nov., and displayed by other canthocamptids (see above) The presence of long slender spinules in the posterior margin of body somites of *Cletocamptus* as defined here, is assumed to be the derived condition.

Posterior cuticular sensillum-bearing socles have been observed only in *C.merbokensis* comb. nov., for which this condition is regarded as apomorphic. All other canthocamptids lack cuticular sensillum-bearing socles and is regarded here as a clear plesiomorphy and is the hypothesized condition in the ancestral Cletocamptinae subfam. nov.

The rostrum is separated from the cephalothorax in species of *Bryocamptus*, *Gulcamptus* Miura, 1969, *Itunella*, *Mesochra*, *Moraria*, *Pordfus* Özdikmen, 2008, *Boreolimella*, *Carolinicola*, *Dahlakocamptus*, *Heteropsyllus*, *Perucamptus*, *Isthmiocaris*, and *Psammocamptus*. It is fused and sometimes reduced in the remaining genera. The well-developed rostrum separated from the cephalothorax is regarded here as the primitive condition for the family, and is assumed to have been retained in the ancestral Cletocamptinae subfam. nov. The condition of the rostrum of *C.gomezi*, which has been described as fused to the cephalothorax (Suárez-Morales et al. 2013) requires confirmation.

The rostrum lacks any spinular ornamentation in the Canthocamptidae; as far as we know, ventral subdistal spinular ornamentation is present only in the Cletocamptinae subfam. nov. for which is a clear autapomorphy and justifies the new subfamily.

As far as we are aware (we could not review all the descriptions for all the species of Canthocamptidae though), the male rostrum is non-dimorphic in most canthocamptids (e.g., Canthocamptinae, Hemimesochrinae and Epactophaninae), and is regarded here as the primitive condition, which was retained in the ancestral Cletocamptinae subfam. nov. The male rostrum is sexually dimorphic in *Cletocamptus* and *Amphibiperita* for which is regarded here as synapomorphic.(it is also sexually dimorphic in *Taurocletodestumenae* Karaytuğ & Huys, 2004 (the male of *T.dubius* (Noodt, 1958) remains unknown), but the significance of its presence in *Cletocamptus* and *T.tumenae* is uncertain). *Cletocamptoides* gen. nov. retained the primitive non-dimorphic male rostrum.

The canthocamptid female antennule can be eight- to five-segmented. Following Huys and Boxshall (1991) oligomerization is the main evolutionary trend in Copepoda, and the eight-segmented female antennule is the most plesiomorphic condition within the Canthocamptidae. The female antennule is six-segmented in the new subfamily, and is regarded here as the condition for the ancestral Cletocamptinae subfam. nov. *Cletocamptusgravihiatus* was described possessing seven-segmented female antennules, but this requires confirmation.

The canthocamptid antenna has undergone fusion of the basis and the first endopodal segment to form an allobasis. The allobasis of canthocamptids is unarmed or equipped with one or two (one basal, one endopodal) abexopodal setae. The latter is regarded here as the most primitive condition of the allobasis. Two abexopodal setae are retained in most species of Cletocamptinae subfam. nov., but some species (*C.gomezi*, *C.feei*, *C.retrogressus*, *C.merbokensis* comb. nov., *C.helobius* comb. nov., *A.neotropica*) underwent loss of one seta, most probably the endopodal element. The antennary exopod of canthocamptids is mostly one-segmented, but the two-segmented condition is present in species of *Bryocamptus*, *Canthocamptus*, *Gulcamptus*, *Maraenobiotus*, *Mesochra*, and *Heteropsyllus*; it is represented by one seta in species of *Stenocaris* and is absent in *Dahmsopottekina* Özdikmen, 2009. The antennary exopod of the Cletocamptinae subfam. nov. is elongate and one-segmented except for *C.feei* for which the exopod was reported as two-segmented (the latter requires confirmation though), *C.chappuisi* whose antennary exopod was reported as represented by one seta as in *C.helobius* comb. nov. and *C.biushelo* sp. nov., and *Amphibiperita* in which the antennary exopod is lost. The antennary exopod of *C.dominicanus* is a minute segment with a single seta. The ancestral antenna of Cletocamptinae subfam. nov. is assumed to possess an allobasis with two abexopodal setae, and a one-segmented exopod; the free endopodal lobe possessed two spines and a slender seta laterally, and three spines and two geniculate setae distally.

The mandibular palp of Podogennonta is composed of a basis with four setae; a one-segmented endopod with three proximal lateral, three subdistal lateral, and three sets of setae fused basally with three, two, and two setae, respectively; and a four-segmented exopod with armature formula 2, 1, 1, 2 (Seifried 2003). Within the Canthocamptidae, *Carolinicola* (Hemimesochrinae) possesses a primitive biramous palp with the basis possessing three setae, the endopod being armed with six elements, and the one-segmented exopod with four setae (Huys and Thistle 1989). The reconstruction of the mandibular palp of the ancestor of the Cletocamptinae subfam. nov. (Fig. 7A) is based on the most plesiomorphic states observed in the different taxa included in the new subfamily. The ancestral mandibular palp (Fig. 7A) of the Cletocamptinae subfam. nov. is assumed to be two-segmented; basis with two setae; endopod one-segmented, separated from the basis, and equipped with three setae, of which innermost shortest; exopod incorporated into the basis but represented by one seta. The ancestral mandibular palp underwent several changes in different lineages. The ancestral palp (Fig. 7A) lost one of the apical setae, and the basis and endopod became fused (Fig. 7B) like in *C.merbokensis* comb. nov. Further losses include one of the basal setae and the exopodal seta (Fig. 7C) like in *C.helobius* comb. nov. and *C.biushelo* sp. nov. On the other hand, the mandibular palp might have lost one basal seta leading the precursor of *Amphibiperita*-*Cletocamptus* (Fig. 7D). In one lineage, the mandibular palp lost the remaining basal seta and the exopodal element (Fig. 7E) resulting in an unarmed basis and a trisetose endopod like in *Amphibiperita*. In another lineage, the mandibular palp of the precursor of *Amphibiperita*-*Cletocamptus* underwent loss of one of the endopodal setae (Fig. 7F) leading the ancestor of *Cletocamptus*. In one lineage, the basis fused into the coxa with the resulting loss of the basal seta, and the exopodal seta became incorporated into the coxa (Fig. 7G) as in *C.assimilis*, *C.axi*, *C.cecsurirensis*, *C.deborahdexterae*, *C.fourchensis*, *C.goenchim*, *C.koreanus*, *C.levis*, *C.nudus*, *C.pilosus*, *C.schmidti*, *C.sinaloensis*, *C.spinulosus*, and *C.tertius*. The inner endopodal seta looks longer than the outer element in *C.nudus*, and this requires confirmation. One of the endopodal setae, most probably the inner short element, became lost leading a minute endopodal segment with one seta only accompanied by the accessory exopodal seta issuing from the coxa like in *C.pilosus* (Fig. 7H). Further loss includes the disappearance of the exopodal seta (Fig. 7I) like in *C.albuquerquensis*, *C.gomezi*, *C.samariensis*, *C.stimpsoni*, and *C.tainoi*. The palp of *C.gomezi* looks somewhat elongate in Suárez-Morales et al. (2013: 213, fig. 3D) and requires confirmation. In another lineage, the exopodal seta became lost (Fig. 7J) like in *C.retrogressus*. Further modifications involve the fusion of the endopod and basis (Fig. 7K) like in *C.confluens*, and reduction of the palp (Fig. 7L) like in *C.dominicanus*. A transposition of the apical short and long setae was observed in the latter species but the significance of this is uncertain.

The maxillulary basis, exopod and endopod are fused into a single unit, making difficult the homologation and correct counting of their setae. A careful and more detailed study of this appendage is pending, but it seems that the coxal endite bears two setae, and the basis, exopod and endopod are represented by three setae each, and this is considered as the ancestral state for the maxillule. On the other hand, a very strong and spinulose ventral element is present in several species of *Cletocamptus*, e.g., *C.assimilis*, *C.axi*, *C.cecsurirensis*, *C.deborahdexterae*, *C.fourchensis*, *C.levis*, *C nudus*, *C.samariensis*, *C.schmidti*, *C.*, and *C.sinaloensis*, but also in *C.helobius* comb. nov., and *C.biushelo* sp. nov., and seems to have appeared independently in different lineages; the ventral seta on the praecoxal arthrite in the other species is visibly slenderer and is regarded here as the plesiomorphic condition found in the ancestral Cletocamptinae subfam. nov. The condition of *C.gomezi* is not conclusive and requires confirmation.

The architecture of the maxilla and maxilliped is rather constant within the new subfamily. The ancestral maxilla is assumed to bear two syncoxal endites; the endopod is assumed to be completely absorbed into the allobasis and represented by three setae. The ancestral maxilliped is subchelate; the syncoxa bears one seta; the basis is unarmed; the endopod is drawn out into a claw with one accompanying seta.

**Table:** 

	P1	P2	P3	P4	P5
EXP	0;1;0,2,2	0;1;1,2,2	0;1;2,2,2	0;1;2,2,2	♀5
					♂4
ENP	0-1;1,2,1	0-1;2,2,1	♀0;2,2,1	0;0,2,0	♀6
			♂dimorphic:		♂3
			0-1;iap;0,2,0		

iap, inner apophysis

The hypothetical ancestral segmentation and armature formulae of P1–P5 is:

The inner setae of P1–P4EXP are assumed to be whip-like in the ancestral Cletocamptinae subfam. nov., i.e., devoid of a comb tip. See Gómez et al. (2017: 350, table 5) for the species of *Cletocamptus* with setae with comb tip. *Cletocamptoidesbiushelo* sp. nov. bears a seta with comb tip on P2–P4 EXP2; the condition of *C.helobius* comb. nov. is assumed to be the same. The inner setae of P2 and P3EXP (P4EXP lack inner setae) of *Cletocamptoidesmerbokensis* comb. nov., and the inner setae of *A.neotropica* are all whip-like. The ancestral P1ENP is assumed to be two-segmented and prehensile. For the justification of the prehensile P1ENP as the plesiomorphic state see George (2020). The ancestral sexual dimorphism of P3ENP is assumed to be expressed in a three-segmented ramus, with inner sinuous slender long apophysis devoid of an arrow-like tip, as seen in all the members of the Cletocamptinae subfam. nov.

The pair of P5 in the females are separated (baseoendopods not fused medially) in all species of Cletocamptinae subfam. nov. The female P5EXP and BENP are fused in all species of Cletocamptinae subfam. nov. except for *A.neotropica* in which the female P5EXP and BENP are separated. The ancestral pair of the female P5 are assumed to be separated; the exopod and the baseoendopod of each leg are assumed to be separated; the exopod is assumed to bear five setae as in most species of the new subfamily (four setae are present only in *A.neotropica*, *C.samariensis*, *C.confluens*, and *C.merbokensis* comb. nov.; three setae are present only in the new species described herein and in *C.helobius* comb. nov.), and the endopodal lobe is equipped with six setae as in most species of the new subfamily (seven setae have been reported for *C.feei* but this requires confirmation; five setae are present in *C.biushelo* sp. nov. and *C.helobius* comb. nov.). The ancestral pair of the male P5 of Cletocamptinae subfam. nov., are assumed to be fused medially as in most species of the new subfamily (the condition of *C.axi*, *C.cecsurirensis*, *C.retrogressus*, and *C.schmidti* is not conclusive), and the exopod and baseoendopod are assumed to be fused but separated by a deep notch as in most species for which the males are known (e.g., *C.deborahdexterae*, *C.stimpsoni*, and *C.sinaloensis*); the endopodal lobe is hypothesized to be armed with three setae as in the males of all the species of Cletocamptinae subfam. nov. for which the males are known; the ancestral exopod is assumed to bear four elements (three elements have been reported only for *A.neotropica*, *C.confluens*, *C.koreanus*, *C.samariensis*, and *C.helobius* comb. nov.).

The female P6 of the ancestral Cletocamptinae subfam. nov. is assumed to bear two setae; the male P6 is assumed to be represented by two articulate unarmed flaps.

The ancestral caudal rami are assumed here to be longer than broad as in some species of *Cletocamptus*, and equipped with seven setae.

### ﻿The genus *Cletocamptus*

The genus *Cletocamptus* was erected by Schmankevitsch (1875) for *C.retrogressus*. The status of several species of the genus can be found in Gómez et al. (2004), Gómez (2005), Gómez et al. (2007), Gómez and Gee (2009), Gómez et al. (2013), and Gómez et al. (2017), and the complete list of species of the genus as redefined here can be found in the generic diagnosis above.

The ancestral Cletocamptinae subfam. nov. is hypothesized to lack somitic constrictions between somites, tapering gradually posteriad. *Cletocamptus* retained the plesiomorphic lack of such somitic constrictions.

*Cletocamptoides* gen. nov. displays the ancestral shape of somites with finely serrated posterior margins (see below), and the long slender spinules displayed by most species of *Cletocamptus* are assumed here to be the apomorphic condition for the posterior margin of body somites. Also, *Cletocamptus* retained the plesiomorphic lack of posterior cuticular sensillum-bearing socles on body somites.

Amongst the species of *Cletocamptus*, only *C.gomezi* has been described with the rostrum fused to the cephalothorax (Suárez-Morales et al. 2013), but this requires further confirmation. In general, the rostrum of *Cletocamptus* retained its plesiomorphic condition, i.e., well-developed and separated from the cephalothorax. However, the rostrum of *Cletocamptus*, as in the other two genera of Cletocamptinae, *Cletocamptoides* gen. nov. and *Amphibiperita*, display the synapomorphic subdistal ventral spinules that are of diagnostic value for the new subfamily. As said above, and as far as we are aware, the male rostrum is sexually dimorphic in *Cletocamptus* and *Amphibiperita* and seems to be non-dimorphic in *Cletocamptoides* gen. nov. The sexually dimorphic rostrum of *Cletocamptus* is deemed to be derived and constitutes a synapomorphy for that genus and *Amphibiperita*. The significance of the dimorphic male rostrum in *Cletocamptus* and *Taurocletodestumenae* is uncertain, but this is probably the result of convergence. The relatively low consistency index of the tree of maximum parsimony confirms homoplasy events that could be related to this convergence process.

*Cletocamptus* retained the six-segmented female antennule hypothesized for the ancestral Cletocamptinae subfam. nov. The seven-segmented female antennule of *Cletocamptusgravihiatus* awaits verification.

*Cletocamptus* retained the ancestral allobasis of the antenna as well as the two abexopodal setae, and only *C.gomezi*, *C.feei*, and *C.retrogressus* lost one of these setae (one seta only is present also in *C.merbokensis* comb. nov., *C.helobius* comb. nov., and *A.neotropica*). *Cletocamptus* also retained the plesiomorphic one-segmented elongate antennary exopod (the two-segmented condition of the antennary exopod of *C.feei* requires to be confirmed) and the architecture of the free endopodal segment as described above for the ancestral Cletocamptinae subfam. nov.

The architecture of the mandibular palp of *Cletocamptus* is variable and underwent considerable reduction as has been detailed above. Briefly, the mandibular palp of the ancestral *Cletocamptus* might have been two-segmented, i.e., basis and endopod separated, being the basis and endopod equipped with one and two setae respectively, and the exopod represented by one seta (Fig. 7F). The ancestral mandibular palp underwent several changes (Fig. 7G–L) across two main lineages, the *retrogressus*-*confluens*-*dominicanus*-lineage (rcd-lineage; Fig. 7J, K, L), and the *assimilis*-*pilosus*-*albuquerquensis*-lineage (apa-lineage; Fig. 7G–I) (see also above). The rcd-lineage is defined upon the synapomorphic loss of the exopodal seta; *C.confluens* and *C.dominicanus* underwent secondary fusion of the basis and endopod which is regarded as synapomorphic for them, and *C.dominicanus* underwent secondary apomorphic reduction of the palp. The apa-lineage is defined here upon the synapomorphic absorption of the basis into the coxa and incorporation of the exopodal seta into the latter. *Cletocamptuspilosus* (Fig. 7H) underwent further loss of one of the endopodal setae which is considered here apomorphic for the species. *Cletocamptusalbuquerquensis*, *C.gomezi*, *C.samariensis*, *C.stimpsoni*, and *C.tainoi* (Fig. 7I) underwent secondary loss of the exopodal seta which is regarded here as synapomorphic for them.

The maxillule is probably the most complicated buccal appendage in Harpacticoida due to the fusion of the basis, endopod and exopod in many taxa, which makes difficult the homologation and correct counting of the setal elements. Pending a more detailed and precise study of this appendage in *Cletocamptus*, the coxal endite possesses two setae, and the basis, the endopod and exopod are fused into one single unit, with three setae representing each of them. On the other hand, a core of species, C.*assimilis*, *C.axi*, *C.cecsurirensis*, *C.deborahdexterae*, *C.fourchensis*, *C.levis*, *C.nudus*, *C.samariensis*, *C.schmidti*, *C.*, and *C.sinaloensis*, share the presence of a very strong spinulose ventral seta on the praecoxal arthrite, which is regarded here as derived within the genus (the condition in *C.gomezi* is inconclusive; a similar seta is present in some species of *Cletocamptoides* gen. nov.). On the contrary, a visibly slenderer seta in the other species of the genus is regarded here as the plesiomorphic condition (a similar seta is present in *C.merbokensis* comb. nov.).

The maxilla and maxilliped retained the plesiomorphic architecture of the Cletocamptinae subfam. nov. as described above.

The P1ENP of *Cletocamptus* is two-segmented and non-prehensile. As said above, this is the derived condition within Canthocamptidae as opposed to the plesiomorphic prehensile endopod (see George 2020). Gómez et al. (2017) gave a list of species of *Cletocamptus* armed with setae with comb tips on P2–P4 EXP2. The presence of such setae is deemed to be derived as opposed to the whip-like setae, but this condition seems to occur widely in the Canthocamptidae.

Sexual dimorphism in *Cletocamptus* is expressed in a variety of modifications. These have been outlined above. The most common modifications in the appendages of the males include the subchirocer antennule, the basis of P1 with one inner distal process, the inner spine on P2 ENP2 with several degrees of modifications, the three-segmented P3ENP with an inner apophysis on second segment and two apical setae on the distal segment, and the fused exopod and baseoendopod of P5. Deviations occur in some species though. The shape of the outer spine on P2 ENP2 varies from slightly modified as in *C.albuquerquensis*, *C.cecsurirensis*, *C.confluens*, *C.dominicanus*, *C.retrogressus*, *C.samariensis*, and *C.tainoi*, to moderately modified as in *C.axi*, *C.deborahdexterae*, *C.fourchensis*, *C.goenchim*, *C.gomezi*, *C.levis*, *C.schmidti*, *C.spinulosus*, *C.stimpsoni*, and *C.tertius*, and strongly modified into a recurved spine as in *C.pilosus*, and *C.sinaloensis*. The inner spine on the male P2 ENP2 is not dimorphic in *A.neotropica* and *Cletocamptoides* gen. nov., and the sexually modified inner spine on P2 ENP2 is regarded here as an apomorphy for *Cletocamptus*. The male P3ENP in most species retained the three-segmented condition with an inner apophysis on second segment which is regarded here as plesiomorphic, but the second and third segments underwent fusion leading a two-segmented ramus with the inner apophysis well-developed and situated medially on second segment as in *C.dominicanus*, or very short and recurved, and situated subdistally on second segment as in *C.albuquerquensis*, *C.chappuisi*, *C.confluens*, and *C.tainoi*. The former division between the second and third segments of the P3ENP is still visible in *C.dominicanus*, which also possesses very long asprothekes (see Gómez et al. 2017). A similar condition of the male P3ENP, two-segmented with inner apophysis medially on second segment, is present also in *A.neotropica*. The P3 ENP2 and ENP3 of *C.albuquerquensis*, *C.chappuisi*, and *C.tainoi* are also fused, but the inner apophysis migrated to a subapical position and underwent extreme reduction, and became recurved. A similar condition is present in *C.helobius* comb. nov. The condition of the male P3ENP of *C.confluens* is unique in that the second segment possesses and additional outer apophysis whose female homologue is uncertain. The pair of male P5 are fused medially in most species, but the exopod and endopod are separated in *C.axi*, *C.cecsurirensis*, *C.retrogressus*, and *C.schmidti*. The significance of this is not clear, but this could be due to character reversal. The male P6 of *Cletocamptus* is an unarmed articulated plate. A small seta has been observed issuing from P6 in some species, but the presence of this seta is most probably due to intraspecific variability. Several sexual modifications are present also in the relative length and shape of P2EXP and/or P3–P4EXP as in *C.retrogressus*, *C.confluens*; *C.albuquerquensis*, *C.samariensis*, *C.spinulosus*, *C.tainoi*, and *C.trichotus*. Similar sexual dimorphism in P4EXP in which the first segment is visibly more robust has been documented for *Amphibiperita* (see Fiers and Rutledge 1990).

### ﻿The genus *Amphibiperita*

The genus *Amphibiperita* has been diagnosed by Fiers and Rutledge (1990). They (Fiers and Rutledge 1990) gave a lengthy discussion on the relationships between *Mesochra* and *Amphibiperita* and justified the creation of the latter for *M.neotropica* Jakobi, 1956. Briefly, Fiers and Rutledge (1990) removed *M.neotropica* from *Mesochra* based on the sexual dimorphism of P4EXP, lack of antennal exopod, reduced mandibular palp, number of setae on the endopods of swimming legs (lack of the outer element on ENP2), and shape of the female genital field. Fiers and Rutledge (1990) commented on the uniqueness of the shape of the sexually dimorphic male P4 EXP1, but similar dimorphism is present in some species of *Cletocamptus*, pointing to a probable sister-group relationship between these two genera, which can be reinforced by the synapomorphic sexually dimorphic rostrum in both genera. Fiers and Rutledge (1990) also commented on the unique lack of the outer subdistal element of P1–P4 ENP2 (Fiers and Rutledge (1990) explicitly included here the P1ENP, but this ramus is prehensile in *Amphibiperita*, with ENP2 being armed with a distal outer claw, a distal inner bipinnate seta, and an inner small slender seta, and in our opinion, the inclusion of P1ENP in Fiers and Rutledge’s (1990) comment is not correct and is most probably a slip of the pen). On the other hand, the subdistal outer element on P2–P4 ENP2 is also missing in *C.merbokensis* comb. nov., being the supernumerary setae in *A.neotropica* the main difference between both species (three, four, and three elements in total in P2–P4 ENP2 in *A.neotropica*, but two, two, and two setae in P2–P4 ENP2 in *M.merbokensis* comb. nov.). Also, the complement armature of P4 ENP2 or only segment in the Cletocamptinae subfam. nov. as defined here is consistently composed of two apical elements only in all but in *A.neotropica*, which possesses one inner additional seta. As noted above, sexual dimorphism in *Amphibiperita* is expressed in the antennule, rostrum, segmentation of the urosome, P5, P6, and more importantly in the shape of the P4 EXP1 and modification of the P3ENP. Some comments on the dimorphic P4 EXP1 and P3ENP, and rostrum were given above. The male P5 of *Amphibiperita* follows the general pattern of *Cletocamptinae* subfam. nov., i.e., exopod and endopod fused and both legs fused medially into an elongate plate with three endopodal and three exopodal setae. On the contrary, the baseoendopod and exopod are separated in the female P5 and is regarded here as primitive within the new subfamily.

Karaytuğ and Huys’ (2004)*Mesochra* group (see above) was proposed for marine and brackish canthocamptids in which the male P6 is represented by unarmed membranous flaps, and the female P6 is represented by one to three setae (*Mesochra*, *Amphibiperita*, *Psammocamptus*, *Bathycamptus*, *Mesopsyllus*, *Isthmiocaris*, *Hemimesochra*, *Poria*, *Perucamptus*, *Pusillargillus*, *Taurocletodes*, and *Parepactophanes*) as opposed to most freshwater canthocamptids in which the male sixth legs bear two or three well-developed setae (Karaytuğ and Huys 2004, and references therein). *Cletocamptus* and *Cletocamptoides* gen. nov. should be added to this list. The significance of the occasional presence of one seta in some species of *Cletocamptus* is still controversial (see above).

### ﻿The genus *Cletocamptoides* gen. nov.

The ancestral Cletocamptinae subfam. nov. lack somitic constrictions between somites (see above). The body shape of *Cletocamptoides* gen. nov. is cletodid-like, i.e., body somites clearly separated by somitic constrictions, and is a probable apomorphy for the new genus. On the other hand, *C.merbokensis* comb. nov. is the only species with integumental sensillum-bearing socles along the posterior margin of pro- and urosomites (except anal somite). However, the presence of integumental sensillum-bearing socles are deemed to be apomorphic for *C.merbokensis* comb. nov., and do not seem to indicate a close relationship with the Cletodidae.

The posterior margin of body somites of most canthocamptid genera can be plain, finely or deeply serrate, or with long slender spinules (see above). The posterior margin of the body somites of *Cletocamptoides* gen. nov. is finely serrate and is regarded here as plesiomorphic within the Cletocamptinae subfam. nov.

The condition of the antennary exopod and the mandibular palp of *C.merbokensis* comb. nov. are the most primitive within *Cletocamptoides* gen. nov. The antennary exopod of *C.merbokensis* comb. nov. is one-segmented and elongate, and bears three setae (one lateral, two distal); the antennary exopod of *C.helobius* comb. nov. and *C.biushelo* sp. nov. is reduced to one minute segment bearing only one seta. Similar antennary exopods are also found in Cletodidae.

The condition of the mandibular palp is more primitive in *C.merbokensis* comb. nov. than in the other two species of the genus. The mandibular palp of *C.merbokensis* comb. nov. possesses five setae; three setae are present in *C.helobius* comb. nov., and *C.biushelo* sp. nov. Besides the supernumerary setal complement in *C.merbokensis* comb. nov. relative to the other two species of the new genus, the basis and exopod are still discernible in the former species, being the inner – basal – extension armed with two setae; the basis of the mandibular palp of *C.helobius* comb. nov. and *C.biushelo* sp. nov. is completely absorbed into the palp but is still discernible by the presence of one inner seta. The exopod of the mandibular palp of *C.merbokensis* comb. nov. is indicated by a small outer protuberance with one seta; the exopod in *C.helobius* comb. nov. and *C.biushelo* sp. nov. underwent complete reduction and is not discernible. The two apical setae on the mandibular palp of these three species are regarded here as endopodal, and judging by their relative lengths, being the inner seta visibly shorter than the outer, both setae are homologous in these three species and in those species with similar armature (see above). The one-segmented condition of the mandibular palp is the most common in the Cletodidae, but the basal, endopodal and/or exopodal setae are still discernible in some species (e.g., *Paracrenhydrosomanormani* Gee, 1999b); the basis and endopod are separated, and the exopod is absorbed into the basis and is represented by a seta in *Pyrocletodes* Coull, 1973 (e.g., *P.coulli* Dinet, 1976); uniramous mandibular palps (basis and endopod separated) without any trace of the exopod are present also in some species of *Paracrenhydrosoma* (e.g., *P.cornuta* Kornev & Chertoprud, 2008, *P.kiai* Song, Dahms, Lee, Ryu & Khim, 2014), and in the monotypic genus *Nannopodella* Monard, 1928.

The unisetose condition of the maxillipedal syncoxa is present in most Cletocamptinae subfam. nov., and it is unarmed only in *C.merbokensis* comb. nov. and *C.helobius* comb. nov. for which is regarded here as apomorphic. Unarmed maxillipedal syncoxae are also present in species of some other canthocamptids e.g., *Attheyella*, *Lessinocamptus*, *Paramoriaropsis* Brancelj, 1991, *Pordfus* Özdikmen, 2008, and *Termomesochra* Itô & Burton, 1980 (Canthocamptinae), and *Stygepactophanes* and *Epactophanes* (Epactophaninae); the maxillipedal syncoxa is unisetose in the Cletodidae (George 2020).

The P1ENP of *Cletocamptoides* gen. nov. displays the derived two-segmented condition (see also George (2020) for a detailed explanation on the plesiomorphic prehensile versus the derived non-prehensile P1ENP in Podogennonta), but the armature complements of P2 and P3 of *C.helobius* comb. nov. and *C.biushelo* sp. nov. display a more primitive trisetose condition of P2–P3 ENP2, relative to the bisetose more derived condition of P2–P3 ENP2 of *C.merbokensis* comb. nov. Fiers and Rutledge (1990) noticed the lack of a subdistal outer element on P3–P4 ENP2 and used that character to differentiate *Amphibiperita* from *Mesochra*. The same condition is present in *C.merbokensis* comb. nov.

One-segmented P4 endopods are present in species of some other canthocamptids, e.g., *Antrocamptus* Chappuis, 1957, *Australocamptus* Karanovic, 2004, *Gulcamptus* Miura, 1969, *Hypocamptus*, *Lessinocamptus* Stoch, 1997, *Morariopsis*, and *Elaphoidella* (Canthocamptinae), *Itunella* and *Isthmiocaris* (Hemimesochrinae), and *Stygepactophanes* Moeschler & Rouch, 1984 and *Epactophanes* (Epactophaninae), but as far as we know from the literature available, it is an elongate and well-developed ramus. Within the new subfamily, a one-segmented P4ENP is present also in *C.dominicanus*, but it is elongated. The P4ENP of *Cletocamptoides* gen. nov. is one-segmented but it has undergone extreme reduction and is an apomorphy, and probable autapomorphy, detected for the new genus.

Both rami of P5 are fused and separated from the supporting somite in the females and males of the genera attributed to the Cletocamptinae subfam. nov., except for the female P5 of *Amphibiperita* whose female P5EXP is separated from the baseoendopod. Both P5 are fused in the males, except for some species of *Cletocamptus* (e.g., *C.axi*, *C.cecsurirensis*, *C.retrogressus* and *C.schmidti*), and *C.helobius* comb. nov. The male of *C.merbokensis* comb. nov. displays the more derived condition of P5, being completely absorbed into the somite, and is considered a potential apomorphy for the species. The male and female P5 of *C.helobius* comb. nov. and *C.biushelo* sp. nov. resembles those of *C.dominicanus*, *C.albuquerquensis*, *C.confluens*, *C.tainoi*, and most probably *C.chappuisi*, in that both rami are hardly discernible and are separated only by a superficial shallow notch. A similar P5 is present also in *Carolinicola* Huys & Thistle, 1989 (Hemimesochrinae). The structure of the cletodid P5 is variable; the endopodal lobe and the basis can be fused or separated, and the exopod can be fused to or separated from the baseoendopod (George 2020).

The *Mesochra* group as defined by Karaytuğ and Huys (2004) (see above) is characterized by the male and the female P6 being represented by unarmed membranous flaps, and by one to three setae, respectively. Besides the twelve genera attributed to this species group by Karaytuğ and Huys (2004) (see above), *C.merbokensis* comb. nov. also belongs to this group. The presence of such male P6 in different lineages could be the result of the artificial nature of the canthocamptid subfamilies, but it is most probably due to convergent evolution. The uni-, bi-, or trisetose condition of the female P6 is widely distributed in the family.

## Supplementary Material

XML Treatment for Cletocamptinae

XML Treatment for Cletocamptus

XML Treatment for Amphibiperita

XML Treatment for Cletocamptoides

XML Treatment for Cletocamptoides biushelo

## References

[B1] Apostolov A (1984) Sur la présence de *Cletocamptusdeitersi* (Richard, 1897) (Copepoda, Harpacticoida) à Cuba. Travaux du Museum d’Histoire Naturelle “Grigore Antipa” 26: 7–10.

[B2] Becker K-H (1979) Eidonomie und taxonomie abyssaler Harpacticoidea (Crustacea, Copepoda) Teil II. Paramesochridae, Cylindropsyllidae und Cletodidae. “Meteor” Forschungsergebnisse Reihe D 31: 1–37.

[B3] Boeck A (1865) Oversigt over de ved Norges Kyster iagttagne Copepoder henhørende til Calanidernes, Cyclopidernes og Harpactidernes Familier. Forhandlinger i Videnskabs-Selskabet i Kristiania 1864: 226–282.

[B4] Boeck A (1872) Nye Slaegter og Arter af Saltvands-Copepoder. Forhandlinger i Videnskabs-Selskabet i Kristiana 1872: 35–60.

[B5] Borutzky EV (1931) Materialien zur Harpacticidenfauna des Baikalsees. II. Zoologischer Anzeiger 93: 263–273.

[B6] Borutzky EV (1952) 3 Fauna SSSR. Rakoobraznye. Harpacticoida presnykh vod. [Fauna of U.S.S.R. Crustacea. Freshwater Harpacticoida]. Izdatel’stvo Akademii Nauk SSSR, Moscow, 424 pp. [in Russian; English translation by Mercado]

[B7] Bowman TE Abele LG (1982) Classification of the recent Crustacea. In: Bliss DE Abele LG (Eds) The Biology of Crustacea 1: Systematics, the fossil record, and biogeography.Academic Press, New York, 1–27.

[B8] Boxshall GA Halsey SH (2004) An introduction to copepod diversity. Vols. I & II. The Ray Society, London, 966 pp.

[B9] Brady GS (1872) Contributions to the study of the Entomostraca. No. VII. A list of the non-parasitic marine Copepoda of the north-east coast of England. Annals and Magazine of Natural History 4: 1–17. 10.1080/00222937208696629

[B10] Brady GS (1880) 2 A monograph of the free and semi-parasitic Copepoda of the British Islands. The Ray Society, London, 182 pp. https://www.biodiversitylibrary.org/item/67810#page/7/mode/1up

[B11] Brady GS (1896) On entomostraca collected in the Solway district and at Seaton Sluice, Northumberland, during the summer of 1894. Natural History Transactions of Northumberland 13: 19–33.

[B12] Brady GS Robertson D (1873) Contributions to the study of the Entomostraca. No. VIII. On marine Copepoda taken in the west of Ireland. Annals and Magazine of Natural History 12: 126–142. 10.1080/00222937308680724

[B13] Brady GS Robertson D (1876) Report on dredgings off the coast of Durham and North Yorkshire in 1874. Report of the British Association for the Advancement of Science held at Bristol in August 1875 1 & 2: 185–199.

[B14] Brancelj A (1991) *Paramorariopsisanae* gen. n., sp. n. and the female of *Ceuthonectesrouchi* Petkovski, 1984 - 2 Interesting Harpacticoids (Copepoda, Crustacea) from Caves in Slovenia (NW Yugoslavia). Stygologia 6: 193–200.

[B15] Brehm V (1936) Mitteilungen von den Forschungsreisen Prof. Rahms. Mitteilung VII. Schlußmitteilung über Cladoceren und Copepoden.-Über den Formenkreis de *Delachauxiellatrigonura* (Ekman). *Macrothrixatahualpa* nov. spec. und *Godetella*. Zoologischer Anzeiger 115: 317–325.

[B16] Brehm V (1965) Bericht über eine unvollendet gebliebene Untersuchung der Argentinischen Kopepodenfauna. Sitzungsberichte der Mathemathisch-naturwissenschaftlichen Klasse, Abteilung 1 174: 1–15. 10.1007/978-3-662-26601-4_1

[B17] Burgess R (2001) An improved protocol for separating meiofauna from sediments using colloidal silica sols. Marine Ecology Progress Series 214: 161–165. 10.3354/meps214161

[B18] Chang CY (2013) A new species of *Cletocamptus*Copepoda (Harpacticoida, Canthocamptidae) from salt marshes in Korea. Animal Systematics, Evolution and Diversity 29: 227–237. 10.5635/ASED.2013.29.3.227

[B19] Chappuis PA (1924) Descriptions préliminaires de copépodes nouveaux de Serbie. Buletinul Societatii de Stiinte din Cluj, România 2: 27–45.

[B20] Chappuis PA (1929a) Die Unterfamilie der Canthocamptinae. Archiv für Hydrobiologie 20: 471–516.

[B21] Chappuis PA (1929b) Révision du genre *Canthocamptus* Westwood (Note peréliminaire). Buletinul Societăţii de Stiinţe din Cluj 4: 41–50.

[B22] Chappuis PA (1930) Notes sur les copépodes. 4. *Antarctobiotuskoenigi* (Pesta). Buletinul Societatii de Stiinte din Cluj 5: 62–64.

[B23] Chappuis PA (1932) Canthocamptinae nouveaux d’Afrique occidentale française. (Descriptions préliminaires). Buletinul Societatii de Stiinte din Cluj 6: 413–420.

[B24] Chappuis PA (1933) Copépodes (première série). Avec l’énumération de tous les copépodes cavernicoles connus en 1931. Archives de Zoologie Expérimentale et Générale 76: 1–57.

[B25] Chappuis PA (1934) Süsswasser Harpacticiden aus dem Hawaiischen inselgebiet. Bulletin de la Société des Sciences de Cluj (Roumanie) 7: 631–635.

[B26] Chappuis PA (1936) Brasilianische ruderfusskrebse (CrustaceaCopepoda), gesammelt von Herrn Dr. Otto Schubart. IV. Mitteilung. Bulletin de la Société des Sciences de Cluj (Roumanie) 8: 450–461.

[B27] Chappuis PA (1957) Les crustacés de la grotte de Gourgue près Montardi (Ariège). Notes Biospéologiques 11: 127–131.

[B28] Claus C (1863) Die frei lebenden Copepoden mit besonderer Berucksichtigung der Fauna Deutschlands, der Nordsee und des Mittelmeeres. Verlag von Wilhelm Engelmann, Leipzig, 230 pp. 10.5962/bhl.title.58676

[B29] Conroy-Dalton S (2001) Systematics and phylogeny of the Ancorabolidae (Copepoda: Harpacticoida). II. Polyphyly of *Polyascophorus* and description of *Arthuricornua*, new genus. Journal of Crustacean Biology 21: 170–191. 10.1163/20021975-99990115

[B30] Coull BC (1973) Meiobenthic Harpacticoida (Crustacea, Copepoda) from the deep sea off North Carolina I. The genera *Hemimesochra* Sars, *Paranannopus* Lang, and *Cylindronannopus* n. g. Transactions of the American Microscopical Society 92: 185–198. 10.2307/3224915

[B31] Daday E (1902) Mikroskopische Süsswasserthiere aus Patagonien. Természetrajzi Füzetek 25: 201–310.

[B32] Delachaux T (1917) Neue Süßwasserharpacticiden aus Südamerika, gesammelt von Herrn Ingenieur E. Godet in den peruanischen Anden. Zoologischer Anzeiger 49: 315–335.

[B33] Dinet A (1976) Sur une nouvelle forme du genre *Pyrocletodes* Coull, 1973 (Copepoda, Harpacticoida) à position systématique incertaine. Bulletin de la Société Zoologique de France 100: 437–442.

[B34] Dussart BH (1974) Contribution à l’étude des Copépodes des eaux douces d’Éthiopie. Bulletin de l’Institut Fondamental d’Afrique Noire 36, sér. A: 92–116.

[B35] Dussart BH Frutos SM (1986) Sur quelques copépodes d’Argentine. 2. Copépodes du Paraná Medio. Revue d’Hydrobiologie Tropicale 19: 241–262.

[B36] Felsenstein J (2005) PHYLIP (Phylogeny Inference Package) version 3.6. Distributed by the author. Department of Genome Sciences, university of Washington, Seattle.

[B37] Fiers F Rutledge P (1990) Harpacticoid copepods associated with *Spartinaalterniflora* culms from the marshes of Cocodrie, Louisiana (Crustacea, Copepoda). Bulletin de l’Institut Royal des Sciences Naturelles de Belgique Biologie: 105–125.

[B38] Fleeger JW (1980) Morphological variation in *Cletocamptus* (Copepoda: Harpacticoida) with description of a new species from Louisiana salt marshes. Transactions of the American Microscopical Society 99: 25–31. 10.2307/3226077

[B39] Fuentes-Reinés JM Zoppi de Roa E Torres R (2015) A new species of *Cletocamptus* Schmankewitsch, 1875 (Crustacea, Copepoda, Harpacticoida) and the description of the male of *C.nudus* from Colombia. Pan-American Journal of Aquatic Sciences 10: 1–18.

[B40] Gee JM (1999a) A new species of *Cletocamptus* Schmankewitsch 1875 (Copepoda; Harpacticoida) from a mangrove forest in Malaysia. Hydrobiologia 412: 143–153.

[B41] Gee JM (1999b) A revision of *Acrenhydrosoma* (Copepoda, Harpacticoida) with the establishment of *Dyacrenhydrosoma* gen. nov. and *Paracrenhydrosoma* gen. nov. and descriptions of two new species. Cahiers de Biologie Marine 40: 337–357.

[B42] George KH (2020) Restructuring the Ancorabolidae Sars (Copepoda, Harpacticoida) and Cletodidae T. Scott, with a new phylogenetic hypothesis regarding the relationships of the Laophontoidea T. Scott, Ancorabolidae and Cletodidae. Zoosstematics and Evolution 96: 455–498. 10.3897/zse.96.51349

[B43] George KH Schminke HK (2003) *Isthmiocarislongitelson* gen. et sp. nov., a strongly derived harpacticoid (Copepoda) from the Magellan region, and its systematic affinities to certain “canthocamptid” taxa. Journal of Crustacean Biology 23: 119–130. 10.1163/20021975-99990321

[B44] Gómez Noguera SE Fiers F (1997) Two new species of *Mesochra* Boeck, 1865 (Copepoda: Harpacticoida) from a costal lagoon in Sinaloa State, Mexico. Bulletin de l’Institut Royal des Sciences Naturelles de Belgique 67: 39–56.

[B45] Gómez S (2005) New species of *Cletocamptus* and a new and fully illustrated record of *C.sinaloensis* (Copepoda: Harpacticoida) from Brazil. Journal of Natural History 39: 3101–3135. 10.1080/17415970500264335

[B46] Gómez S (2020) On some new species of Stenheliinae Brady, 1880 (Copepoda: Harpacticoida: Miraciidae) from north-western Mexico, with the proposal of *Lonchoeidestenhelia* gen. nov. Zookeys 987: 41–79. 10.3897/zookeys.987.52906 33223885 PMC7666071

[B47] Gómez S Fleeger JW Rocha-Olivares A Foltz D (2004) Four new species of *Cletocamptus* Schmankewitsch, 1875, closely related to *Cletocamptusdeitersi* (Richard, 1897) (Copepoda: Harpacticoida). Journal of Natural History 38: 2669–2732. 10.1080/0022293031000156240

[B48] Gómez S Gee JM (2009) On four new species of *Cletocamptus* Shmankevich, 1875 (Copepoda: Harpacticoida) from inland waters of Argentina. Journal of Natural History 43: 2853–2910. 10.1080/00222930903374171

[B49] Gómez S Gerber R Fuentes-Reinés JM (2017) Redescription of *Cletocamptusalbuquerquensis* and *C.dominicanus* (Harpacticoida: Canthocamptidae*incertae sedis*), and description of two new species from the US Virgin Islands and Bonaire. Zootaxa 4272: 301–359. 10.11646/zootaxa.4272.3.1 28610279

[B50] Gómez S Ingole B Sawant M Singh R (2013) *Cletocamptusgoenchim* sp. nov., a new harpacticoid (Copepoda: Harpacticoida) from India. Proceedings of the Biological Society of Washington 126: 259–275. 10.2988/0006-324X-126.3.259

[B51] Gómez S Scheihing R Labarca P (2007) A new species of *Cletocamptus* (Copepoda: Harpacticoida) from Chile and some notes on *Cletocamptusaxi* Mielke, 2000. Journal of Natural History 41: 39–60. 10.1080/00222930601141476

[B52] Gurney R (1927) Prof. Labbés Copepod ‘Allomorphs’. Nature 120: 295–296. 10.1038/120295b0

[B53] Gurney R (1932) The British fresh-water Copepoda. II. Harpacticoida. The Ray Society, London, 336 pp. 10.5962/bhl.title.82138

[B54] Hamond R (1973) The harpacticoid copepods (Crustacea) of the saline lakes in Southeast Australia, with special reference to the Laophontidae. Records of the Australian Museum 28: 393–420. 10.3853/j.0067-1975.28.1973.406

[B55] Hamond R (1987) Non-marine copepods of Australia. I. Canthocamptidae of the genus *Canthocamptus* Westwood s. lat. and *Fibulacamptus*, gen. nov., and including the description of a related new species of *Canthocamptus* from New Caledonia. Invertebrate Systematics 1: 1023–1047. 10.1071/IT9871023

[B56] Herbst VH V (1960) Copepoden (Crustacea, Entomostraca) aus Nicaragua und Südperu. Gewasser und Abwasser 27: 27–54.

[B57] Herrick CL (1894) Microcrustacea from New Mexico. Zoologischer Anzeiger 18: 40–47.

[B58] Huelsenbeck JP Ronquist F (2001) MRBAYES: Bayesian inference of phylogeny. Bioinformatics 17: 754–755. 10.1093/bioinformatics/17.8.754 11524383

[B59] Huys R (2009) Unresolved cases of type fixation, synonymy and homonymy in harpacticoid copepod nomenclature (Crustacea: Copepoda). Zootaxa 2183: 1–99. 10.11646/zootaxa.2183.1.1

[B60] Huys R Boxshall GA (1991) Copepod evolution. The Ray Society, London, 468 pp. 10.4319/lo.1993.38.2.0478

[B61] Huys R Conroy-Dalton S (2006) Revision of the genus *Evansula* T. Scott, 1906 (Copepoda, Harpacticoida, Cylindropsyllidae) with a description of three new species. Zoological Journal of the Linnean Society 147: 419–472. 10.1111/j.1096-3642.2006.00227.x

[B62] Huys R Gee J Moore C Hamond R (1996) Marine and brackish water harpacticoid copepods. Part 1. In: Barnes R, Crothers J (Eds) Synopses of the British fauna (New Series). Published for The Linnean Society of London and The Estuarine and Coastal Sciences Association by Field Studies Council Shrewsbury, London, 352 pp.

[B63] Huys R Kihara TC (2010) Systematics and phylogeny of Cristacoxidae (Copepoda, Harpacticoida): a review. Zootaxa 2568: 1–38. 10.11646/zootaxa.2568.1.1

[B64] Huys R Thistle D (1989) *Bathycamptuseckmani* gen. et spec. nov. (Copepoda, Harpacticoida) with a review of the taxonomic status of certain other deepwater harpacticoids. Hydrobiologia 185: 101–126. 10.1007/BF00010809

[B65] Itô T Burton JJS (1980) A new genus and species of the family Canthocamptidae (Copepoda, Harpacticoida) from a hot spring at Dudun Tua, Selangor, Malaysia. Zoologische Jahrbücher, Abteilung für Systematik 107: 1–31.

[B66] Jakobi H (1956) Novas espécies de Harpacticoidea (Copepoda-Crustacea) provenientes de regiões de água salobra da costa São Paulo-Paraná. (Neue Harpacticoiden-Arten (Copepoda-Crustacea) aus den Brackwassergebieten der Küste São Paulo-Paraná). Dusenia 7: 159–171.

[B67] Karanovic T (2004) Subterranean copepods (Crustacea, Copepoda) from arid Western Australia. Crustaceana Monograph, Brill, Leiden, 366 pp. 10.1163/9789047412779

[B68] Karaytuğ S Huys R (2004) Taxonomic position of and generic distinction between *Parepactophanes* Kunz, 1935 and *Taurocletodes* Kunz, 1975 (Copepoda, Canthocamptidae*incertae sedis*), with description of a new species from the Black Sea. Zoological Journal of the Linnean Society 140: 469–486. 10.1111/j.1096-3642.2003.00101.x

[B69] Kiefer F (1929) Eine neue Harpacticoiden-Form aus Südafrika: *Cletocamptustrichotus* n. sp. Zoologischer Anzeiger 84: 21–23.

[B70] Kiefer F (1933) Über zwei Arten der Gattung *Cletocamptus* Schmankewitsch (CopepodaHarpacticoida). Zoologischer Anzeiger 105: 141–144.

[B71] Kiefer F (1934) Neue Ruderfußkrebse von der Insel Haiti. Zoologischer Anzeiger 108: 227–233.

[B72] Kiefer F (1936) Freilebende Süß- und Salzwassercopepoden von der Insel Haiti. Mit einer Revision der Gattung *Halicyclops* Norman. Archiv für Hydrobiologie 30: 263–317.

[B73] Kiefer F (1957) Freilebende Ruderfußkrebse (CrustaceaCopepoda) aus einigen ostanatolischen Seen. Zoologischer Anzeiger 159: 25–33.

[B74] Klie W (1929) Die CopepodaHarpacticoida der südlichen und westlichen Ostsee mit besonderer Berücksichtigung der Sandfauna der Kieler Bucht. Zoologische Jahrbücher, Abteilung fur Systematik, Ökologie und Geographie der Tiere 57: 329–386.

[B75] Kornev PN Chertoprud EC (2008) Copepod crustaceans of the order Harpacticoida of the White Sea: morphology, systematics, ecology. Biology Faculty MSU (Ed.) Tovarishchestvo Nauchnikh Izdanii KMK, Moscow, 379 pp.

[B76] Kunz H (1935) Zur Ökologie der Copepoden Schleswig-Holsteins und der Kieler Bucht. Schriften des Naturwissenschaftlichen Vereins für Schleswig-Holstein 21: 84–132.

[B77] Kunz H (1975) Harpacticoiden (Crustacea, Copepoda) aus dem Küstengrundwasser der französischen Mittelmeerküste. Zoologica Scripta 3: 257–282. 10.1111/j.1463-6409.1974.tb00822.x

[B78] Labbé A (1927) Contributions à l’étude de allélogénèse. III-memoire. L’histoire naturelle des copépodes des Marais Salants du Croisic. Essai de phylogénie expérimentale. Archives de Zoologie Expérimentale et Générale 66: 135–290.

[B79] Labbé MA (1926) Les Rhyncoceratinae, groupe nouveau de copépodes harpacticides (note préliminaire). Bulletin de la Société Zoologique de France 51: 441–444.

[B80] Lang K (1936) Beiträge zur Kenntnis der Harpacticiden. 6. Bemerkungen über die Familie der Ameiridae Monard. Zoologischer Anzeiger 114: 133–136.

[B81] Lang K (1944) Monographie der Harpacticiden (vorläufige Mitteilung). Almqvist & Wiksells Boktryckeri AB, Uppsala, 39 pp.

[B82] Lang K (1948) Monographie der Harpacticiden. Vols. I & II. Nordiska Bokhandeln, Stockholm, 1682 pp.

[B83] Lang K (1965) CopepodaHarpacticoidea from the Californian Pacific coast. Kungliga Svenska Vetenskapsakademiens Handlingar (4)10: 1–560. http://www.vliz.be/imis/imis.php?refid=78667

[B84] Löffler H (1961) Beiträge zur Kenntnis der Iranischen Binnen-gewässer II. Regional-limnologische studie mit besonderer Berücksichtigung der Crustaceenfauna. Internationale Revue der Gesamten Hydrobiologie 46: 309–406. 10.1002/iroh.19610460304

[B85] Löffler H (1963) Zur Ostrakoden- und Copepodenfauna Ekuadors. Archiv für Hydrobiologie 59: 196–234.

[B86] Loftus WF Reid JW (2000) Copepod (Crustacea) emergence from soils from Everglades marshes with different hydroperiods. Journal of Freshwater Ecology 15: 515–523. 10.1080/02705060.2000.9663774

[B87] Mielke W (1975) Systematik der Copepoda eines Sandstrandes der Nordseeinsel Sylt. Mikrofauna des Meeresbodens 52: 1–134.

[B88] Mielke W (2000) Two new species of *Cletocamptus* (Copepoda: Harpacticoida) from Galapagos, closely related to the cosmopolitan *C.deitersi*. Journal of Crustacean Biology 20: 273–284. 10.1163/20021975-99990039

[B89] Miura Y (1969) Results of the speleological survey in South Korea 1966. XIV. Subterranean harpacticoid copepods of South Korea. Bulletin of the National Science Museum, Tokyo, Series A, Zoology 12: 241–254.

[B90] Moeschler P Rouch R (1984) Un nouveau genre de Canthocamptidae (Copepoda, Harpacticoidea) des eaux souterraines de Suisse. Revue Suisse de Zoologie 91: 959–972. 10.5962/bhl.part.81592

[B91] Monard A (1927) Synopsis universalis generum Harpacticoidarum. Zoologische Jahrbücher, Abteilung für Systematik 54: 139–176.

[B92] Monard A (1928) Les harpacticoïdes marins de Banyuls. Archives de Zoologie Expérimentale et Générale 67: 259–443.

[B93] Monard A (1935) Étude sur la faune des harpacticoïdes marins de Roscoff. Travaux de la Station Biologique de Roscoff 13: 5–88.

[B94] Mrázek A (1893) Beitrag zur Kenntniss der Harpacticidenfauna des Süsswassers. Zoologische Jahrbücher. Abteilung für Systematik, Geographie und Biologie der Tiere 7: 89–130.

[B95] Noodt W (1958) Die CopepodaHarpacticoidea des Brandungsstrandes von Teneriffa (Kanarische Inseln). Abhandlungen der Mathematisch-Naturwissenschaftlichen Klasse, Akademie der Wissenschaften und der Literatur in Mainz 1958: 53–116.

[B96] Oliveira LP Krau L Miranda ASA (1971) Plâncton poluído da Guanabara com copépodos *Cletocamptus* e rotíferos Rotaria. Archivos do Museu Nacional do Rio de Janeiro 54: 55–56.

[B97] Özdikmen H (2008) Nomenclatural changes for nine crustacean genera (Crustacea: Copepoda). Munis Entomology and Zoology 3: 265–275.

[B98] Özdikmen H (2009) Substitute names for two genera of Harpacticoida (Crustacea: Copepoda). Munis Entomology & Zoology 4: 297–298.

[B99] Pesta O (1932) Krebstiere oder Crustacea. I: Ruderfüsser oder Copepoda. 3. Unterordnung: Harpacticoida (1. und 2. Hälfte). In: Dahl F (Ed.) Die Tierwelt Deutschlands und der Angrenzenden Meeresteile nach Ihren Merkmalen und nach Ihrer Lebensweise.Verlag von Gustav Fischer, Jena, 1–164.

[B100] Philippi A (1840) Zoologische Bemerkungen. Archiv für Naturgeschichte 6: 181–195. from: https://www.biodiversitylibrary.org/page/7203160

[B101] Por F (1960) *Mesopsyllusatargatis* n. g., n. sp., ein neuer Harpacticoide (Crustacea, Copepoda) aus dem Schwarzen Meere. Travaux du Museum d’Histoire Naturelle “Grigore Antipa” 2: 177–181.

[B102] Por FD (1968) Copepods of some land-locked basins on the islands of Entedebir and Nocra (Dahlak Archipelago, Red Sea). Sea Fishery Research Station in Haifa Bulletin 49: 37–41.

[B103] Por FD (1986) A re-evaluation of the family Cletodidae Sars, Lang (Copepoda, Harpacticoida). Proceedings of the Second International Conference on Copepoda, Ottawa, Canada, 13–17 August 1984, Schriever G, Schminke HK, Shih C-t (Eds) Syllogeus 58: 420–425.

[B104] Ranga Reddy Y Radhakrishna Y (1979) A new record of *Cletocamptusdeitersi* (Richard, 1895) (Copepoda: Harpacticoida) from India. Current Science 48(1): 45–45.

[B105] Richard J (1897) Entomostracés de l’Amérique du Sud, recuellis par MM. U. Deiters, H. Von Ihering, G. W. Müller et C. O. Poppe. Mémoires de la Société Zoologique de France 10: 263–301.

[B106] Ringuelet RA (1958a) Los crustáceos copépodos de las aguas continentales en la República Argentina. Sinopsis sistemática. Contribuciones Científicas de la Facultad de Ciencias Exactas y Naturales de la Universidad de Buenos Aires, Serie Zoología 1: 1–120.

[B107] Ringuelet RA (1958b) Primeros datos ecológicos sobre copépodos dulciacuícolas de la República Argentina. Physis 21: 14–31.

[B108] Ringuelet RA (1960) Datos de fecundidad en copépodos dulciacuícolas. Physis 21: 316–317.

[B109] Ringuelet RA (1962) Rasgos faunísticos de las reservas naturales de la Provincia de Buenos Aires. Physis 23: 83–91.

[B110] Ringuelet RA Moreno I Feldman E (1967) El zooplancton de las lagunas de la Pampa deprimida y otras aguas superficiales de la llanura bonaerense (Argentina). Physis 27: 187–200.

[B111] Rohal M Thistle D Easton EE (2016) Extraction of metazoan meiofauna from muddy deep-sea samples: operator and taxon effects on efficiency. Journal of Experimental Marine Biology and Ecology 502: 105–110. 10.1016/j.jembe.2017.01.006

[B112] Ruber E Gilbert A Montagna PA Gillis G Cummings E (1994) Effects of impounding coastal salt marsh for mosquito control on microcrustacean populations. Hydrobiologia 292–293: 497–503. 10.1007/BF00229977

[B113] Sars GO (1906) CopepodaHarpacticoida Parts XV & XVI Diosaccidae (concluded), Canthocamptidae (part). An account of the Crustacea of Norway with short descriptions and figures of all the species 5: 173–196.

[B114] Sars GO (1909) CopepodaHarpacticoida. Cletodidae (concluded), Anchorabolidae, Cylindropsyllidae, Tachidiidae (part). An account of the Crustacea of Norway with short descriptions and figures of all the species 5: 305–336.

[B115] Sars GO (1911) CopepodaHarpacticoida Parts XXXIII & XXXIV Supplement (continued). An account of the Crustacea of Norway with short descriptions and figures of all the species 5: 397–420.

[B116] Sars GO (1920) Copepoda Supplement. Parts V & VI. Harpacticoida (continued). An account of the Crustacea of Norway with short descriptions and figures of all the species 7: 53–72.

[B117] Schmankevitsch VI (1875) Nekotoryya rakoobraznyya solyano-ozernykh’ i presnykh vod’ i otneshenie ikh’ k’ srede. Some Crustacea of salt and freshwater lakes, and their relation to the surrounding environment. Zapiski Novorossiiskago Obshchestva Estestvoispytatelei (= Mémoires de la Société des Naturalistes de la Nouvelle Russie, Odessa) 3: 1–391.

[B118] Schmeil O (1894) Einige neue Harpacticiden-Formen des Süsswassers. Zeithschrift für Naturwissenschaften 67: 341–350.

[B119] Schriever G (1983) New Harpacticoidea (Crustacea, Copepoda) from the North-Atlantic Ocean. III. New species of the family Cletodidae. Meteor Forschungsergebnisse Reihe D Supplement: 65–83.

[B120] Scott T (1892) III Additions to the fauna of the Firth of Forth. Part IV. Annual Report of the Fishery Board for Scotland 10: 244–272.

[B121] Scott T (1894) II Additions to the fauna of the Firth of Forth. Part VI. Annual Report of the Fishery Board for Scottland 12: 231–271.

[B122] Scott T (1902) Notes on gatherings of Crustacea collected by the fishery steamer “Garland”, and the steam trawlers “Star of Peace” and “Star of Hope”, of Aberdeen, during the year 1901. Annual Report of the Fishery Board for Scotland. 20: 447–484.

[B123] Scott T (1904) On some new and rare Crustacea from the Scottish seas. Reports of the Fishery Board for Scotland 23: 141–153.

[B124] Scott T (1906a) A catalogue of the land, fresh-water and marine Crustacea found in the basin of the River Forth and its estuary. II: The Ostracoda, Copepoda, and Cirripedia. Proceedings of the Royal Physical Society of Edinburgh 16: 267–386. 10.5962/bhl.title.53706

[B125] Scott T (1906b) LXII. Notes on British Copepoda: change of names. Annals and Magazine of Natural History 7: 458–466. 10.1080/00222930608562556

[B126] Scott T Scott A (1893) On some new or rare Scottish Entomostraca. Annals and Magazine of Natural History 6: 210–215. 10.1080/00222939308677499

[B127] Seifried S (2003) Phylogeny of Harpacticoida (Copepoda): revision of “Maxillipedasphalea” and Exanechentera. Cuvillier Verlag, Göttingen, 259 pp.

[B128] Shen C-J (1956) On a collection of Copepoda from Chinghai Province and inner Mongolia. Acta Zoologica Sinica 8: 1–12. [In Chinese]

[B129] Shen C-J Sung T (1963) Notes on Copepoda collected from Shigatze and Gyangtse regions in Tibet, China. Acta Zoologica Sinica 15: 79–97. [In Chinese, with English sumary]

[B130] Sitjar CC (1988) Crustáceos del arroyo Naposta Grande (Provincia de Buenos Aires, Argentina). Spheniscus 6: 63–72.

[B131] Smirnov SS (1946) Novye vidy CopepodaHarpacticoida iz severnogo ledovitogo okeana (New species of Copepoda-Harpacticoida) from the northern Arctic Ocean). Trudy Dreif. Eksped. Glav. na ledokol. Parokh. “G. Sedov” 1937–1940: 231–263. [in Russian]

[B132] Song SJ Dahms HU Lee CR Ryu J Khim JS (2014) A new species of *Paracrenhydrosoma* (Copepoda: Harpacticoida: Cletodidae) from a subtidal muddy bottom of southern Korea, with a key to the species of *Acrenhydrosoma*-complex. Journal of the Marine Biological Association of the United Kingdom 94: 981–991. 10.1017/S0025315414000289

[B133] Stĕrba O (1968) Neue Harpacticoidea (Crustacea, Copepoda) aus dem asiatischen Teil der Paläarktis. Zoologischer Anzeiger 180: 50–68.

[B134] Stoch F (1997) A new genus and two new species of Canthocamptidae (Copepoda, Harpacticoida) from caves in northern Italy. Hydrobiologia 350: 49–61. 10.1023/A:1003072813906

[B135] Suárez-Morales E Barrera-Moreno O Ciros-Pérez J (2013) A new species of *Cletocamptus* Schmankewitsch, 1875 (Crustacea, Copepoda, Harpacticoida) from a high altitude saline lake in Central Mexico. Journal of Limnology 72: 313–325. 10.4081/jlimnol.2013.e25

[B136] Suárez-Morales E Reid JW Iliffe TM Fiers F (1996) Catálogo de los copépodos (Crustacea) continentales de la Península de Yucatán, México. Comisión Nacional para el Conocimiento y Uso de la Biodiversidad (CONABIO) and El Colegio de la Frontera Sur (ECOSUR), Unidad Chetumal, Mexico City, 296 pp.

[B137] Tai AY Song YZ (1979) Harpacticoida Sars, 1903. In: Shen C-J, Ai-Yun T, Chong-Zhou Z, Zhi-Ying L, Da-Xiang S, Guo-Xiao C (Eds) Freshwater Copepoda. Fauna Sinica, Crustacea. Science Press, Beijing, 450 pp.

[B138] Wells JBJ (2007) An annotated checklist and keys to the species of CopepodaHarpacticoida (Crustacea). Zootaxa 1568: 1–872. 10.11646/zootaxa.1568.1.1

[B139] Westwood JO (1836) *Cyclops* (Muller). In: Partington CF (Ed.) The British Cyclopaedia of Natural History.Orr & Smith, London, 227–228.

[B140] Willey A (1930) Harpacticoid Copepoda from Bermuda. Part I. Annals and Magazine of Natural History 6: 81–114. 10.1080/00222933008673192

[B141] WoRMS [Editorial Board] (2021) World Register of Marine Species.

[B142] Zamudio-Valdéz JA (1991) Los copépodos de vida libre (Crustacea, Maxillopoda) del Valle de Cuatro Ciénegas, Coahuila, México. Autonomous University of Nuevo León, Monterrey, Mexico, 107 pp.

